# Loss of IL-10 signaling in macrophages limits bacterial killing driven by prostaglandin E2

**DOI:** 10.1084/jem.20180649

**Published:** 2019-12-05

**Authors:** Subhankar Mukhopadhyay, Eva Heinz, Immacolata Porreca, Kaur Alasoo, Amy Yeung, Huei-Ting Yang, Tobias Schwerd, Jessica L. Forbester, Christine Hale, Chukwuma A. Agu, Yoon Ha Choi, Julia Rodrigues, Melania Capitani, Luke Jostins-Dean, David C. Thomas, Simon Travis, Daniel Gaffney, William C. Skarnes, Nicholas Thomson, Holm H. Uhlig, Gordon Dougan, Fiona Powrie

**Affiliations:** 1 Wellcome Trust Sanger Institute, Hinxton, Cambridge, UK; 2 Medical Research Council Centre for Transplantation, Peter Gorer Department of Immunobiology, King's College London, London, UK; 3 Translational Gastroenterology Unit, Experimental Medicine Division, Nuffield Department of Clinical Medicine, University of Oxford, John Radcliffe Hospital, Oxford, UK; 4 Swiss Precision Dignostics Development Company Limited, Bedford, UK; 5 Dr. von Hauner Children’s Hospital, Ludwig-Maximilians-University of Munich, Munich, Germany; 6 Division of Infection and Immunity, Cardiff University, Cardiff, UK; 7 The Kennedy Institute of Rheumatology, University of Oxford, Oxford, UK; 8 Department of Medicine, University of Cambridge, University of Cambridge School of Clinical Medicine, Cambridge, UK; 9 The Jackson Laboratory for Genomic Medicine, Farmington, CT; 10 Department of Pathogen Molecular Biology, London School of Hygiene and Tropical Medicine, London, UK; 11 Department of Paediatrics, University of Oxford, John Radcliffe Hospital, Oxford, UK

## Abstract

Loss of IL-10 signaling in macrophages (Mφs) leads to inflammatory bowel disease (IBD). Induced pluripotent stem cells (iPSCs) were generated from an infantile-onset IBD patient lacking a functional *IL10RB* gene. Mφs differentiated from IL-10RB^−/−^ iPSCs lacked IL-10RB mRNA expression, were unable to phosphorylate STAT3, and failed to reduce LPS induced inflammatory cytokines in the presence of exogenous IL-10. IL-10RB^−/−^ Mφs exhibited a striking defect in their ability to kill *Salmonella enterica* serovar Typhimurium, which was rescuable after experimentally introducing functional copies of the *IL10RB* gene. Genes involved in synthesis and receptor pathways for eicosanoid prostaglandin E2 (PGE2) were more highly induced in IL-10RB^−/−^ Mφs, and these Mφs produced higher amounts of PGE2 after LPS stimulation compared with controls. Furthermore, pharmacological inhibition of PGE2 synthesis and PGE2 receptor blockade enhanced bacterial killing in Mφs. These results identify a regulatory interaction between IL-10 and PGE2, dysregulation of which may drive aberrant Mφ activation and impaired host defense contributing to IBD pathogenesis.

## Abstract (precis)

Cytokines and lipid mediators are key regulators of inflammation; but how they are mechanistically linked is poorly understood. Here, Mukhopadhyay et al. show a novel regulation between cytokine IL-10 and lipid mediator PGE2 that functionally connects them to intestinal inflammation.

## Introduction

The inflammatory bowel diseases (IBDs) encompassing Crohn’s disease and ulcerative colitis are complex chronic inflammatory conditions of the gastrointestinal tract. Alterations in intestinal barrier function, host defense, and immune regulation may lead to aberrant host microbial interactions and chronic intestinal inflammation (Maloy and Powrie, 2011). Mouse models identified IL-10 as a critical cytokine in the maintenance of intestinal homeostasis (Kole and Maloy, 2014; Kühn et al., 1993; Moore et al., 2001) through limiting the activation state of macrophages (Mφs; Bogdan et al., 1991; Smythies et al., 2005). Thus, mice lacking IL-10 can develop severe and spontaneous enterocolitis (Kühn et al., 1993).

IL-10 signals via a heterodimeric receptor complex of the IL-10 receptor α (*IL10RA*) and β (*IL10RB*), exerting an anti-inflammatory effect involving phosphorylation of the transcription factor STAT3 (Hutchins et al., 2013; Hutchins et al., 2012; O’Farrell et al., 1998). Myeloid cell–specific loss of IL-10 receptor α (Shouval et al., 2014a; Zigmond et al., 2014), IL-10 receptor β (Donnelly et al., 2004; Kotenko et al., 1997; Spencer et al., 1998), or STAT3 (Kobayashi et al., 2003) all lead to spontaneous colitis in mice, suggesting that IL-10 signaling in myeloid cells is critical for the maintenance of intestinal homeostasis. In the absence of IL-10 signaling, the resident microbiota drives excessive MyD88-dependent TLR signaling and uncontrolled Mφ activation, contributing to intestinal pathology (Hoshi et al., 2012; Rakoff-Nahoum et al., 2006; Redhu et al., 2017).

Compelling evidence in support of an involvement of IL-10 in human intestinal inflammation comes from clinical observations that rare loss-of-function mutations in the *IL10*, *IL10RA*, or *IL10RB* genes lead to very-early-onset or infantile IBD with severe phenotypes (Glocker et al., 2010; Glocker et al., 2009; Glocker et al., 2011; Moran et al., 2013). In addition, IBD genome-wide association studies (GWAS) have identified common polymorphisms in the IL-10 pathway as increased risk factors for adult-onset polygenic IBD (Ellinghaus et al., 2016; Jostins et al., 2012). While a protective role of IL-10 is relatively well established in the context of IBD and other inflammatory diseases (Shouval et al., 2014b), its role in other traits, including susceptibility to infections is less well understood (Couper et al., 2008; Peñaloza et al., 2016). Several studies have indicated that IL-10 protects the host during infection by limiting pathogen-induced immune pathologies (Couper et al., 2008), but other studies have shown that IL-10 can directly inhibit microbial killing by phagocytes (Fleming et al., 1999; Lee et al., 2011; Oswald et al., 1992), compromising host defense (Redford et al., 2011). In addition, a range of pathogens has been shown to hijack the IL-10 pathway to subvert the host immune response (Avdic et al., 2013; Redpath et al., 2001; Sing et al., 2002a; Sing et al., 2002b).

To study the impact of IL-10 on the inflammatory and microbicidal activities of Mφs and how alterations in IL-10 signaling may contribute to IBD, we generated an induced pluripotent stem cell (iPSC) line from an IBD patient harboring a homozygous mutation in the *IL10RB* gene predicted to introduce a premature stop codon resulting in nonfunctional protein. This patient’s iPSC-derived Mφs are unresponsive to IL-10 and exhibit a dysregulated inflammatory cytokine response to bacterial stimuli such as LPS and *Salmonella enterica* serovar Typhimurium *(S.* Typhimurium). Despite their hyperactivated phenotype, IL-10RB^−/−^ Mφs exhibited a defect in their ability to control the intracellular growth of *S.* Typhimurium, a phenotype we link here to the overproduction of the lipid mediator prostaglandin E2 (PGE2). These data identify a novel reciprocal regulatory loop between IL-10 and PGE2, active in Mφs, that may contribute to IBD pathology.

## Results

### IL-10RB^−/−^ and control Mφs exhibit comparable phenotypes

**Figure 1.**
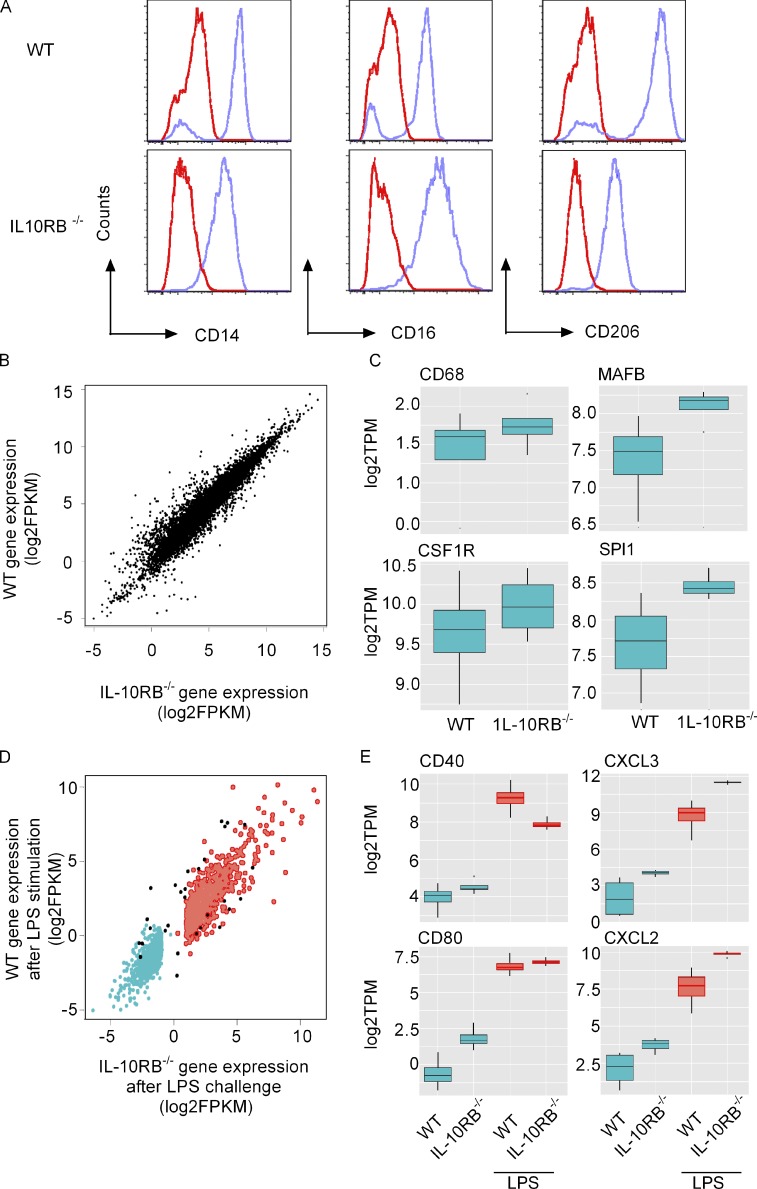
**IL-10RB**^**−/−**^** Mφs develop normally and exhibit a normal transcriptional signature.** Mφs were differentiated from IL-10RB^−/−^ (*n* = 3) and control iPSCs (*n* = 4). **(A)** Surface expression of CD14, CD16, and CD206 were assessed by flow cytometry. Representative histograms from three independent experiments show surface expression of antigen in control fpdj_3 (upper panel) and IL-10RB^−/−^ (lower panel) Mφs. Blue lines indicate specific antibody staining, and red lines indicate staining by isotype-matched controls. **(B)** Average log2FPKM values for all expressed genes in IL-10RB^−/−^ (*n* = 3) and control Mφs (*n* = 4) were evaluated by RNASeq and are presented in a scatter plot. **(C)** Relative mRNA expression of selected Mφ surface markers (CD68 and CSF1R) and transcription factors (MAFB and SPI1) in unstimulated WT (*n* = 4) and IL-10RB^−/−^ (*n* = 3) Mφs are reported as normalized log2TPM values. **(D)** Genes that are significantly induced (red) or repressed (blue) after 6-h LPS stimulation in both WT (*n* = 4) and IL-10RB^−/−^ (*n* = 3) Mφs are presented. Genes that show significantly different expression between control and IL-10RB^−/−^ Mφs after LPS stimulation are shown in black. **(E)** Relative mRNA expression of selected known LPS-responsive costimulatory molecules (CD40 and CD80) and chemokine genes (CXCL2 and CXCL3) were compared between WT (*n* = 4) and IL-10RB^−/−^ (*n* = 3) Mφs after LPS stimulation, and normalized log2TPM values are presented. Statistical significance in B–D was based on FDR <0.05 and fold-change >2. FPKM, fragment per kilobase million.

**Figure S1.**
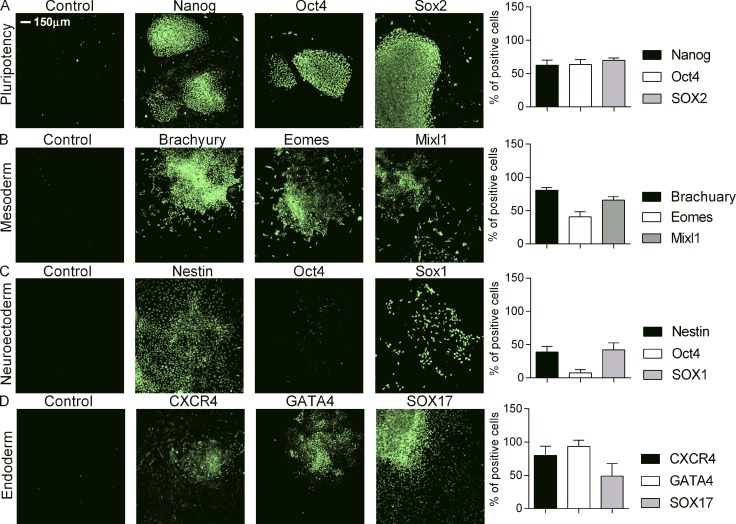
**Patient-derived IL-10RB^−/−^ iPSCs differentiate normally into three germ layers.** IL-10RB^−/−^ iPSCs were either maintained in pluripotency media or cultured in specific differentiation media promoting differentiation into three germ layers: endoderm, mesoderm, and neuroectoderm. Cells were stained with specific antibodies against markers for pluripotency (Nanog, Oct4, and SOX2), mesoderm (Brachyury, EOMES, MIXL1), neuroectoderm (Nestin, SOX1, and SOX2), and endoderm (SOX17, CXCR4, and GATA4) and costained with nuclear dye DAPI (not shown). The expression level of each antigen was analyzed in a Cellomics array scan; the scale bar (2 mm = 150 µm) is the same for all panels. Representative images showing immunofluorescense staining for each marker are shown in green. The percentage of positive cells for each antigen is shown in the corresponding bar diagram. Corresponding bar diagram represents percentage of positive cells for each antigen. Data are shown from at least triplicate wells are presented as means ± SD and are representative of at least two independent experiments.

Skin fibroblasts from a previously reported infantile-onset IBD patient harboring a homozygous loss-of-function splice site mutation in the *IL10RB* gene (Engelhardt et al., 2013) were reprogrammed to induced pluripotency and are henceforth referred to as IL-10RB^−/−^ iPSCs. As controls, we used four independent human iPSC lines (HIPSI0114i-kolf_2, HPSI0813-fpdj_3, HPSI0314i-bubh_1, and HPSI0713i-uimo_1) from unrelated healthy individuals obtained from the Human Induced Pluripotent Stem Cell Initiative (http://www.hipsci.org/; Agu et al., 2015; Kilpinen et al., 2017). The patient-derived iPSCs showed normal characteristics when propagated under specific conditions (Fig. S1). The IL-10RB^−/−^ and the four control iPSC lines were differentiated into Mφs (Alasoo et al., 2015; Hale et al., 2015) with very similar phenotypes in terms of morphology and expression of markers. The IL-10RB^−/−^ and control fpdj_3 Mφs showed comparable surface expression of CD14, CD16, and CD206 by flow cytometry (Fig. 1 A). Similarly, the WT and IL-10RB^−/−^ Mφs also displayed an overall comparable global gene expression profile in the naive state (Fig. 1 B), including similar levels of mRNA expression for Mφ-associated lineage markers (CD68 and CSF1R) and transcription factors (SPI1 and MAFB; Fig. 1 C). The global gene expression was also comparable between WT and IL-10RB^−/−^ Mφs after LPS stimulation (Fig. 1 D), including comparable increases in mRNA levels of known LPS-inducible costimulatory molecules (CD40 and CD80) and the chemokine genes (CXCL3 and CXCL5; Fig. 1 E).

### IL-10RB^−/−^ Mφs are unresponsive to IL-10

**Figure 2.**
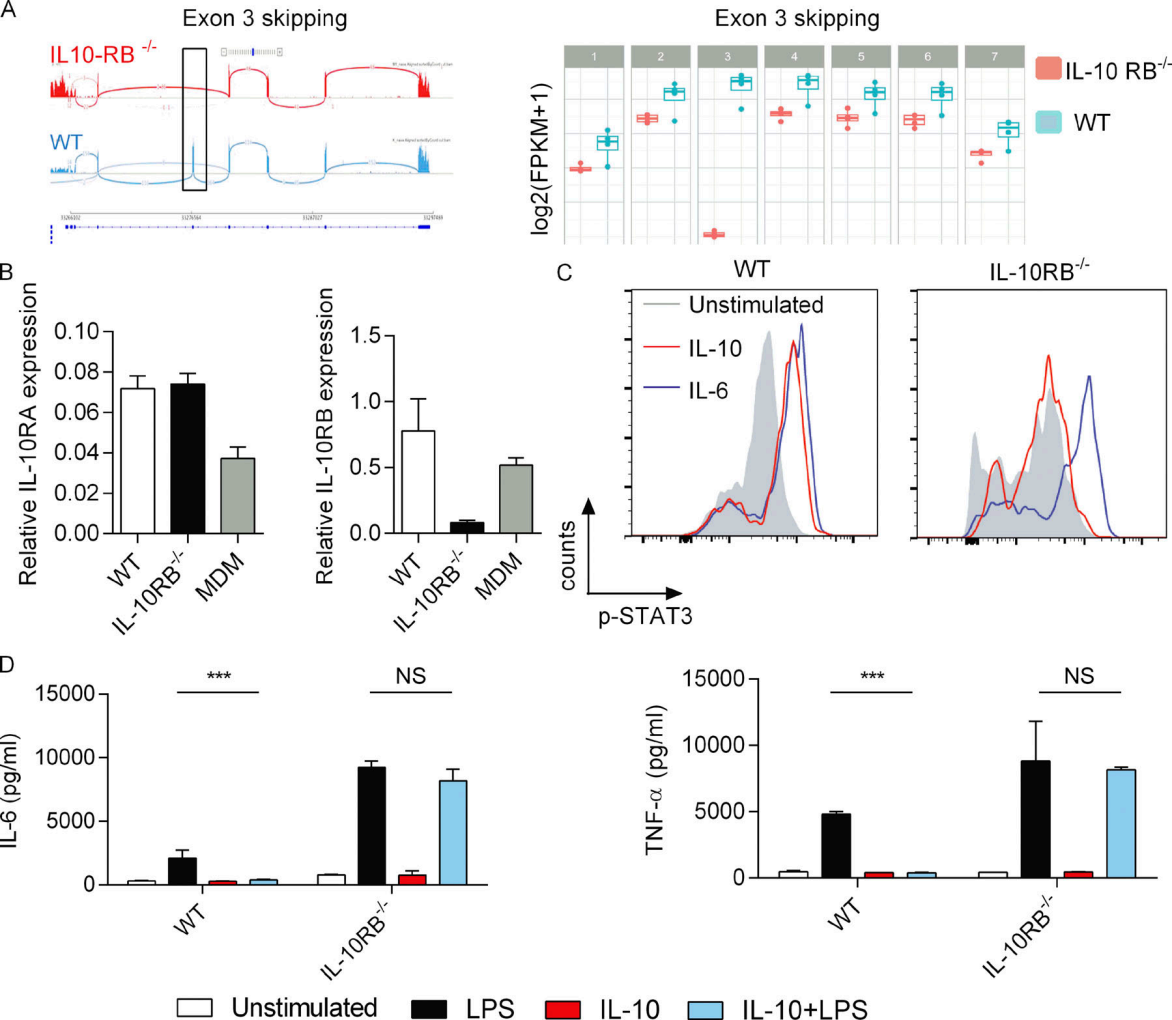
**IL-10RB**^**−/−**^** Mφs are unresponsive to IL-10. (A)** RNASeq read depth across the IL-10RB gene body plotted for control fpdj_3 (blue) and IL-10RB^−/−^ (red) Mφs (left panel), with *IL10RB* gene structure in the panel beneath each plot. The black box indicates the read count from exon 3. The right panel shows normalized read counts of each of the exons of the IL-10RB gene in WT (*n* = 4) and IL-10RB^−/−^ (*n* = 3) Mφs. **(B)** The relative expressions of IL-10RA and IL-10RB mRNA were compared between WT (*n* = 3) and IL-10RB^−/−^ (*n* = 3) Mφs and monocyte-derived Mφs (MDMs; *n* = 3) using gene-specific Taqman RT-qPCR probes. **(C)** IL-10RB^−/−^ and control fpdj_3 Mφs were stimulated with 20 ng/ml of rhIL-10 or IL-6 for 15 min, and expression of phospho-STAT3 (pY705) was measured by flow cytometry after staining with a specific antibody. **(D)** IL-6 and TNF-α levels were measured by ELISA in supernatants of IL-10RB^−/−^ and control kolf_2 Mφs (*n* = 3) prestimulated overnight with 20 ng/ml rhIL-10 or left unstimulated and then challenged with 2 ng/ml LPS for 6 h in the presence of IL-10. Data in all panels are representative of at least three independent experiments. Data in B and D are reported as means ± SD of at least triplicate wells of each condition. Two-way ANOVA with Tukey’s multiple comparisons test was used to assess statistical significance. ***, P < 0.001.

**Figure S2.**
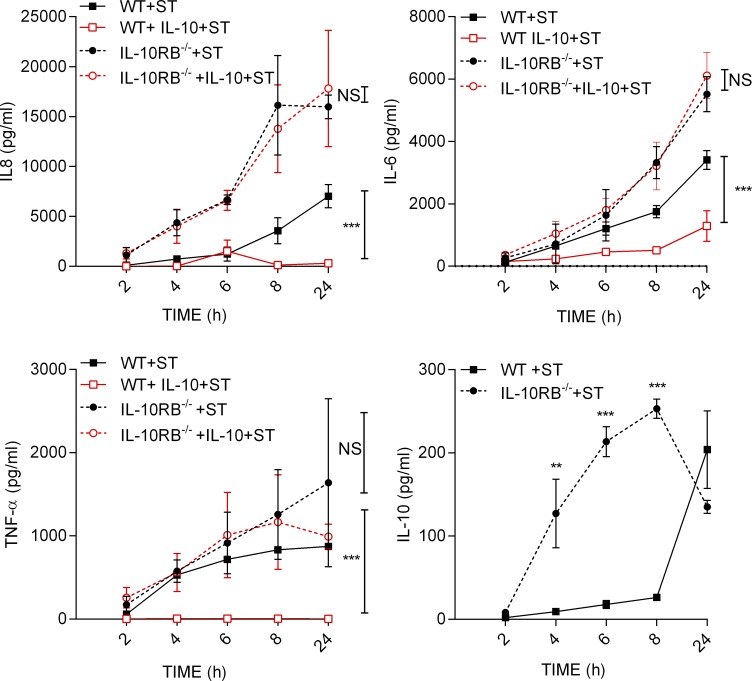
**Cytokine production under IL-10 stimulation and *S.* Typhimurium infection.** IL-6, IL-8, TNF-α, and IL-10 levels were measured in the supernatants of IL-10RB^−/−^ (*n* = 3) and control kolf_2 Mφs (*n* = 3) prestimulated overnight with 20 ng/ml rhIL-10 or left unstimulated and then infected with *S*. Typhimurium (MOI 1) in the presence or absence of IL-10; after 1 h of incubation, cells were washed three times with PBS to remove extracellular bacteria, and fresh medium without antibiotics was added (IL-10 was kept in appropriate wells). After incubation at the indicated time points, 25 µl of medium was harvested and stored at −80°C for cytokine analysis and replaced with the same volume of medium for the subsequent time points. Concentrations of indicated cytokines in harvested supernatants were analyzed by a Luminex assay kit according to the manufacturer’s instructions. Data shown in all panels are from at least triplicate wells, are presented as means ± SD, and are representative of at least three independent experiments. Two-way ANOVA with Tukey’s multiple comparisons test was performed using GraphPad software to assess statistical significance. ***, P < 0.001; **, P < 0.01.

The patient homozygous splice site mutation IVS311G>C located in the exon-intron 3 boundary is predicted to be an exon-skipping variant that leads to a premature stop codon and consequently a truncated, nonfunctional protein (Engelhardt et al., 2013). This prediction was confirmed by comparing exon read counts between IL-10RB^−/−^ and control Mφs after RNA sequencing (RNASeq). Indeed, significant numbers of reads from exon 3 were observed in control Mφs, whereas no detectable exon 3 complementary reads were observed in IL-10RB^−/−^ Mφs (Fig. 2 A). The quantitative PCR (RT-qPCR) analysis showed a significantly decreased IL-10RB mRNA expression in the IL-10RB^−/−^ Mφs compared with control fpdj_3 Mφs or primary monocyte-derived Mφs obtained from healthy donors (Fig. 2 B), suggesting that exon 3 skipping may cause mRNA instability most likely due to nonsense-mediated decay (Brogna and Wen, 2009; Hug et al., 2016). Next, we assessed the impact of the *IL10RB* gene mutation on IL-10 signaling. Both IL-10 and IL-6 induce robust phosphorylation of STAT3, but these two cytokines use distinct receptor complexes for intracellular signaling (Taga and Kishimoto, 1997). Comparison of STAT3 phosphorylation between control fpdj_3 and IL-10RB^−/−^ Mφs after IL-10 or IL-6 stimulation by flow cytometry revealed that IL-10 stimulation induced robust STAT3 phosphorylation in control but not in IL-10RB^−/−^ Mφs. By contrast, IL-6 stimulation induced robust STAT3 phosphorylation in both control fpdj_3 and IL-10RB^−/−^ Mφs (Fig. 2 C), suggesting a selective IL-10 signaling defect in IL-10RB^−/−^ Mφs. Furthermore, both control and IL-10RB^−/−^ Mφs expressed comparable levels of IL-10RA mRNA (Fig. 2 B), indicating that any IL-10–specific defect in STAT3 phosphorylation in IL-10RB^−/−^ Mφs was not due to a defect in *IL10RA* gene expression. The functional consequences of the mutation were further investigated by measuring IL-10–mediated suppression of an LPS-induced inflammatory cytokine response. Exogenous IL-10 inhibited LPS-mediated TNF-α and IL-6 induction in control but not in IL-10RB^−/−^ Mφs (Fig. 2 D), consistent with experiments performed in primary leukocytes from this patient (Engelhardt et al., 2013). Similarly, the kinetics of cytokine production between WT fpdj_3 Mφs and IL-10RB^−/−^ Mφs were compared after exposure to live *S.* Typhimurium. The IL-10RB^−/−^ Mφs produced higher amounts of the pro-inflammatory cytokines IL-8, TNF-α, and IL-6 but also strikingly higher amounts of IL-10 compared with control fpdj_3 Mφs in a time-dependent manner (Fig. S2). Again, exogenous IL-10 suppressed such *S.* Typhimurium–induced cytokine response in control Mφs but not in IL-10RB^−/−^ Mφ (Fig. S2).

### IL-10RB^−/−^ Mφs are defective in bacterial killing

**Figure 3.**
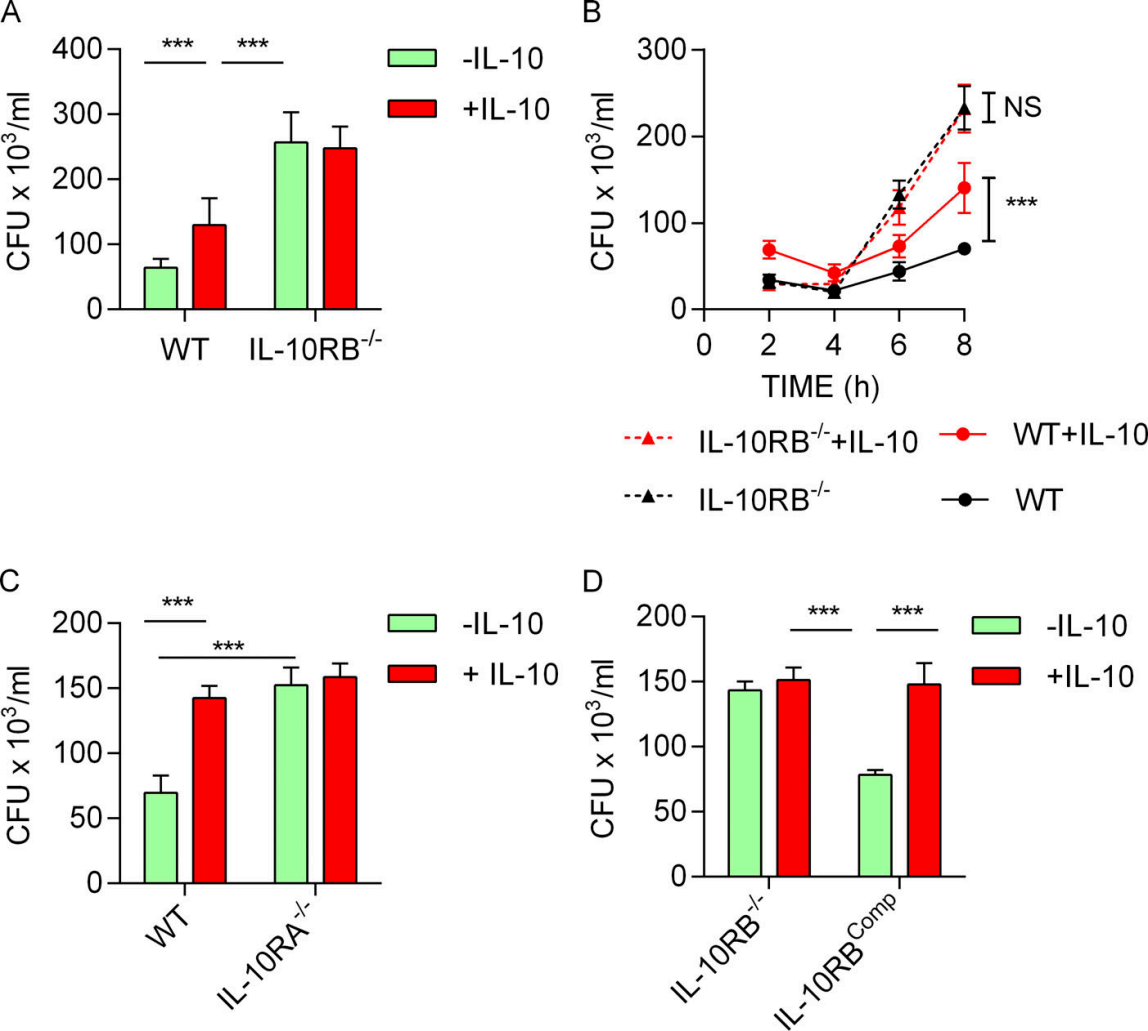
**Loss of IL-10 signaling impairs *S.* Typhimurium killing in Mφs. (A)** Bacterial survival measured by gentamicin protection assay and reported as CFU/ml in kolf_2 and IL-10RB^−/−^ Mφs (*n* = 4) prestimulated with 20 ng/ml rhIL-10 or left unstimulated and infected with 10 MOI of *S.* Typhimurium SL1344 (pssaG:GFP); survival was measured at 5 h. **(B)** The time course of *S.* Typhimurium survival within kolf_2 and IL-10RB^−/−^ Mφs (*n* = 4) in the presence or absence of IL-10 is shown. **(C)** A comparison of survival of *S.* Typhimurium between IL-10RA^−/−^ and control kolf_2 Mφs in the presence or absence of IL-10 is presented at 5 h. **(D)** Comparison of *S.* Typhimurium survival/replication between IL-10RB^−/−^ and IL-10RB^comp^ Mφs (*n* = 4). Data shown in all panels are from quadruplicate wells, are presented as means ± SD, and are representative of at least three independent experiments. Two-way ANOVA with Tukey’s multiple comparisons test was performed using GraphPad software to assess statistical significance. ***, P < 0.001.

**Figure S3.**
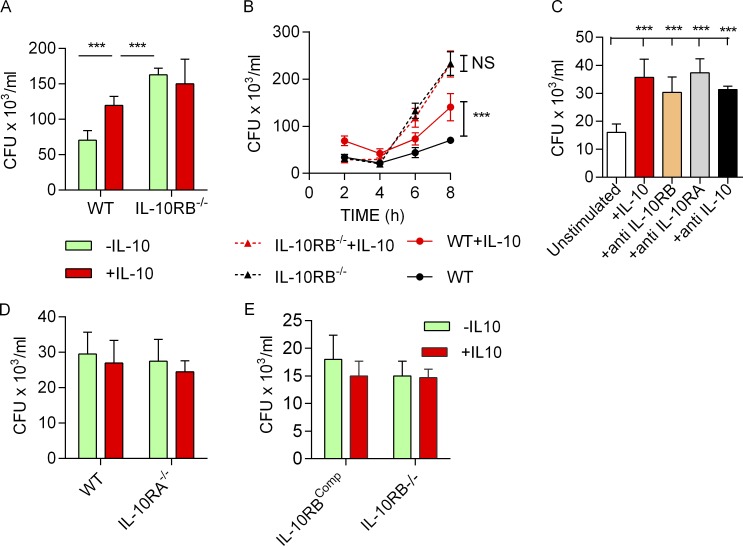
**IL-10 signaling blockade leads to reduced bacterial killing in Mφs. (A)** Bacterial survival measured by gentamicin protection assay and reported as CFU/ml in fpdj_3 and IL-10RB^−/−^ Mφs (*n* = 4) prestimulated with 20 ng/ml rhIL-10 or left unstimulated and infected with 10 MOI of *S.* Typhimurium SL1344 (pssaG:GFP); survival was measured at 5 h. **(B)** Time course of *S.* Typhimurium survival within fpdj_3 and IL-10RB^−/−^ Mφs (*n* = 4) in the presence or absence of IL-10 is shown. **(C)** The survival of *S.* Typhimurium within fpdj_3 control Mφs in the presence or absence of blocking antibodies against IL-10, IL-10RA, and IL-10RB (*n* = 4) is presented as bar diagrams. **(D and E)** Initial uptake of *S.* Typhimurium between WT control kolf_2 Mφs and IL-10RA^−/−^ Mφs (*n* = 4; D) and between IL-10RB^−/−^ and IL-10RB^comp^ Mφs (*n* = 4; E) were compared at 1-h incubation with bacteria before adding gentamicin. Data shown in all panels are from at least quadruplicate wells, are presented as means ± SD, and are representative of at least three independent experiments. Two-way ANOVA with Tukey’s multiple comparisons test was performed using GraphPad software to assess statistical significance. ***, P < 0.001.

**Figure S4.**
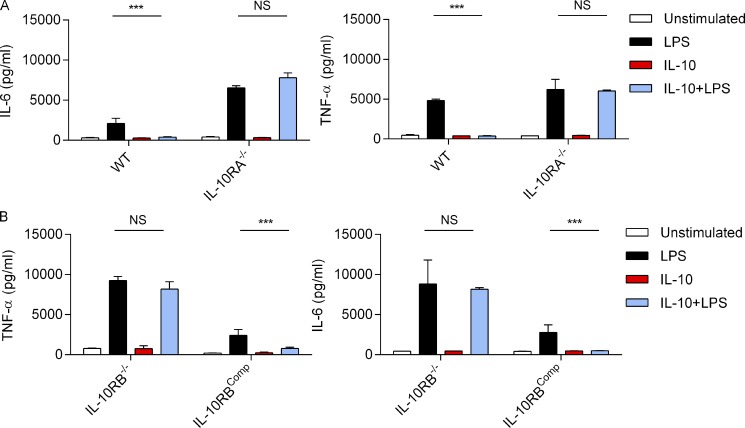
**Experimental deletion of IL-10RA leads to an IL-10 unresponsive phenotype, and genetic complementation of IL-10RB**^**−/−**^** rescues the IL-10 unresponsive phenotype. (A)** IL-6 and TNF-α levels were measured by ELISA in supernatants of IL-10RA^−/−^ and control kolf_2 Mφs (*n* = 3) prestimulated overnight with 20 ng/ml rhIL-10 or left unstimulated and then challenged with 2 ng/ml LPS for 6 h in the presence of IL-10. **(B)** IL-6 and TNF-α levels were measured by ELISA in supernatants of IL-10RB^−/−^ and IL-10RB^comp^ Mφs (*n* = 3) prestimulated overnight with 20 ng/ml rhIL-10 or left unstimulated and then challenged with 2 ng/ml LPS for 6 h in the presence of IL-10. Data shown in all panels are from at least triplicate wells, are presented as means ± SD, and are representative of at least three independent experiments. Two-way ANOVA with Tukey’s multiple comparisons test was performed using GraphPad software to assess statistical significance. ***, P < 0.001.

Next, we investigated whether the loss of IL-10 signaling impacts microbial killing in IL-10RB^−/−^ Mφs. To assess this, IL-10RB^−/−^ and control kolf_2 Mφs were infected with *S.* Typhimurium in the presence or absence of exogenous IL-10, and the amount of intracellular bacteria was assessed by a gentamicin protection assay. Consistent with previous reports (Fleming et al., 1999; Lang et al., 2002b; Lee et al., 2011), we show that IL-10 pretreatment reduced intracellular killing of *S.* Typhimurium in control kolf_2 Mφs but not in IL-10RB^−/−^ Mφs (Fig. 3 A). Unexpectedly, unstimulated IL-10RB^−/−^ Mφs exhibited a striking defect in bacterial killing compared with control kolf_2 Mφs. Kinetic analysis showed that this difference was more prominent between control and IL-10RB^−/−^ Mφs at later time points (Fig. 3 B). The relative reduction in bacterial killing in IL-10RB^−/−^ Mφs was also observed when compared with fpdj_3 control Mφs (Fig. S3, A and B). Since the control and IL-10RB^−/−^ Mφs originated from unrelated individuals, we wanted to exclude the possibility that the observed effect was a consequence of differences in their genetic background as opposed to a specific loss of IL-10 signaling. To address this issue, first, a bacterial killing assay was performed in control Mφs in the presence or absence of blocking antibodies against IL-10, IL-10RA, or IL-10RB. Blockade of IL-10/IL-10R signaling significantly reduced bacterial killing in control Mφs (Fig. S3 C). In addition, we performed genetic complementation of the IL-10RB^−/−^ iPSC line by introducing a functional copy of the *IL10RB* gene using a transcription activator-like effector nuclease (TALEN)–based method and designated this line as IL-10RB^comp^. Separately, the *IL10RA* gene was knocked out (IL-10RA^−/−^) in the control kolf_2 iPSC line using CRISPR/Cas9 methods (Forbester et al., 2018; Yeung et al., 2017). The IL-10RB^comp^ and IL-10RA^−/−^ iPSCs were differentiated into Mφs and functionally validated in an IL-10–mediated LPS suppression assay. As expected, IL-10 failed to suppress LPS-induced IL-6 and TNF-α secretion in IL-10RA^−/−^ Mφs compared with their isogenic WT controls (Fig. S4 A). By contrast, exogenous IL-10 suppressed LPS-induced IL-6 and TNF-α secretion in the IL-10RB^comp^ Mφs, but not in the original IL-10RB^−/−^ Mφs (Fig. S4 B). Bacterial killing was assessed in IL-10RB^−/−^, IL-10RB^comp^, IL-10RA^−/−^, and control kolf_2 Mφs. IL-10RA^−/−^ Mφs showed significantly reduced bacterial killing compared with the isogenic kolf_2 Mφs (Fig. 3 C). Additionally, defective killing of IL-10RB^−/−^ Mφs was rescued in IL-10RB^comp^ Mφs (Fig. 3 D). Considering IL-10 can affect both phagocytic uptake and intracellular killing within Mφs, we set out to clarify this issue. Bacterial uptake between IL-10RA^−/−^ and IL-10RB^−/−^ Mφs and their corresponding isogenic control Mφs was compared after initial incubation with bacteria before adding gentamicin. No difference in bacterial uptake was observed between IL-10R–deficient Mφs and their WT controls (Fig. S3 D). These findings provide strong evidence that the enhanced bacterial count observed in IL-10RA^−/−^ and IL-10RB^−/−^ Mφs compared with their controls is due to reduced intracellular killing.

### IL-10 modulates subsets of LPS-regulated genes

**Figure 4.**
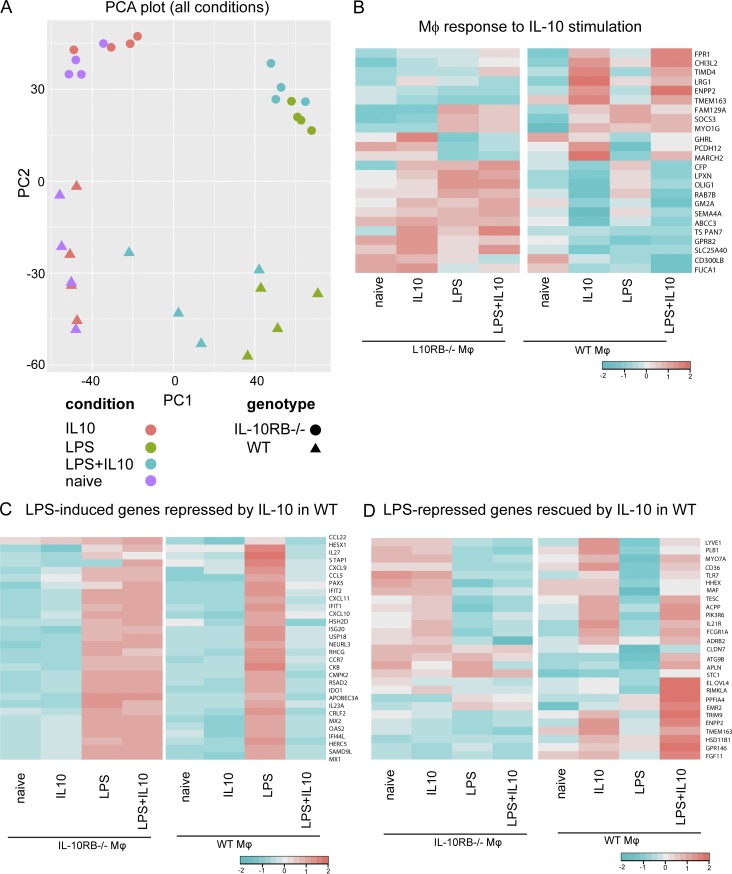
**IL-10 selectively inhibits the LPS-mediated transcriptional response in Mφs. (A)** PC analysis (PCA) was performed on all expressed genes in control Mφs (*n* = 4) and IL-10RB^−/−^ Mφs (*n* = 3) stimulated with either 20 ng/ml rhIL-10 (overnight) or 2 ng/ml LPS for 6 h, or IL-10 prestimulation followed by a 6-h LPS stimulation (in the presence of IL-10). **(B)** Heat maps showing average gene expression levels of ∼25 genes significantly deregulated after IL-10 stimulation in control Mφs (*n* = 4) but not regulated in IL-10RB^−/−^ Mφs (*n* = 3; Table S3). **(C)** Heat maps showing expression levels of selected LPS-induced genes that are down-regulated in the IL-10 plus LPS condition in control Mφs but remained unchanged in IL-10RB^−/−^ Mφs (IL-10–repressed genes). **(D)** Heat maps showing expression levels of selected LPS-repressed genes whose expression was significantly reversed in IL-10 plus LPS treatment in control Mφs but not in IL-10RB^−/−^ Mφs (IL-10–rescued genes). Only the top most significant genes are presented in C and D (full lists of affected genes are presented in Tables S4 and S5, respectively). Statistical significance in B–D was based on FDR <0.05 and fold-change >2.

**Figure S5.**
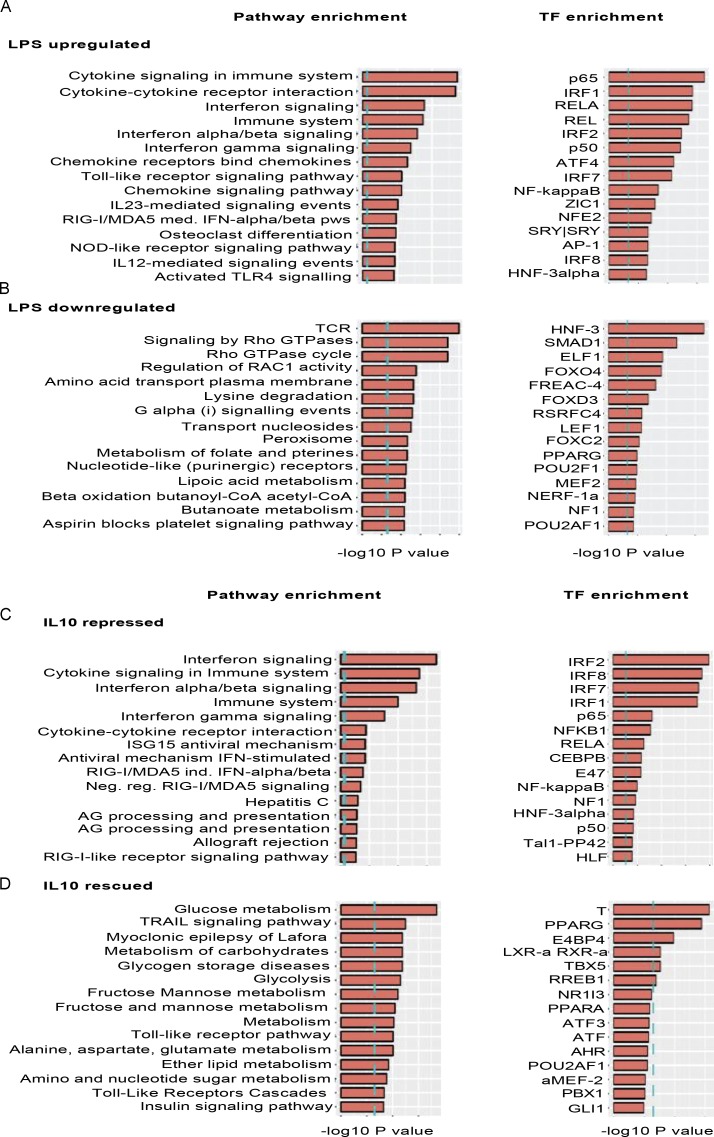
**Pathways and transcription factor enrichment analyses of LPS-responsive genes.** Differentially expressed genes between unstimulated and LPS-stimulated conditions were calculated from the average gene expression values from IL-10RB^−/−^ (*n* = 3) and control Mφs (*n* = 4) using DESeq analysis, based on FDR <0.05 and fold-change >2. **(A)** Pathway enrichment analysis, transcription factor (TF) target gene enrichment analysis, and Gene Ontology term enrichment analysis were performed using corresponding InnateDB analysis tools on all genes that were significantly up-regulated after LPS treatment. The top 15 terms for each analysis are shown based on the P value ranking. **(B)** The same enrichment analysis performed on all genes significantly down-regulated after LPS stimulation in both Mφs and the top 15 terms for each analysis are shown based on the P value ranking. **(C)** Pathway enrichment analysis and transcription factor target gene enrichment analysis performed on LPS-inducible genes that are significantly repressed in the IL-10 plus LPS condition. **(D)** Pathway enrichment analysis and transcription factor target gene enrichment analysis on LPS down-regulated genes that are rescued in the IL-10 plus LPS treatment condition in control Mφs but not in IL-10RB^−/−^ Mφs using the same criteria.

We compared the transcriptomes of IL-10RB^−/−^ and control Mφs in the following conditions: (i) unstimulated; (ii) overnight IL-10 stimulation; (iii) 6-h LPS stimulation; and (iv) overnight IL-10 prestimulation followed by 6-h LPS challenge. With the application of principal component (PC) analysis, the first component (PC1) clearly separated unstimulated and LPS-stimulated control and IL-10RB^−/−^ Mφs, accounting for ∼39.86% of the variance. By contrast, IL-10–stimulated samples exhibited an almost identical expression pattern in both control and IL-10RB^−/−^ Mφs (Fig. 4 A), indicating that IL-10 by itself did not induce a strong transcriptional response even in WT Mφs. Interestingly, control Mφs stimulated with IL-10 plus LPS clustered closer to unstimulated samples, suggesting a partial inhibition of the LPS response by IL-10. By contrast, IL-10RB^−/−^ Mφs did not show this intermediate clustering when stimulated with a combination of IL-10 and LPS (Fig. 4 A), indicating that IL-10 had no obvious effect on these cells. WT Mφs were separated from each other as well as from IL-10RB^−/−^ Mφs along PC2, with 22.53% variance irrespective of stimulation. This is in part driven by inherent differences in genetic background among individual iPSC lines as well as specific differences in the IL-10RB genotype. Comparison of the IL-10 response beween WT and IL-10RB^−/−^ Mφs revealed that only a small number of genes (∼25) were selectively regulated by IL-10 in WT Mφs, and these remained unchanged in IL-10RB^−/−^ Mφs (Fig. 4 B and Table S3), indicating that IL-10 stimulation alone induced a rather modest transcriptional response even in WT Mφs. These IL-10–regulated genes included SOCS3 and MARCH1 (Fig. 4 B) with known anti-inflammatory functions (Croker et al., 2003; Galbas et al., 2017; Hunt et al., 2012; Mittal et al., 2015; Wong et al., 2006). Similarly, WT and IL-10RB^−/−^ Mφs showed an almost identical transcriptional response to LPS stimulation with ∼1,050 up-regulated (Table S1) and ∼950 down-regulated genes (Table S2) in both Mφs. Only ∼20 LPS-regulated genes were significantly different between WT and IL-10RB^−/−^ Mφs, but these also showed the same direction of regulation. Pathway enrichment analysis of LPS-induced genes showed significant enrichment of pathways involved in cytokine signaling, interferons, chemokines, IL-23, and TLR signaling. Similarly, transcription factor enrichment analysis of LPS-induced genes revealed significant enrichment of targets for interferon regulatory factors (IRFs) and NF-κB components (Fig. S5 A). By contrast, the LPS down-regulated genes included Rho GTPases, amino acid and lipid metabolism, β oxidation pathways, and targets of transcription factors including SMAD1, FOXO, and PPARγ (Fig. S5 B). Comparison of LPS-stimulated Mφs with LPS plus IL-10 treatment showed that IL-10 selectively repressed the expression of ∼29% of LPS up-regulated genes (Fig. 4 C) and selectively rescued ∼8% of LPS down-regulated genes (Fig. 4 D) in WT Mφs but had no effect in IL-10RB^−/−^ Mφs (Fig. 4, C and D), indicating that IL-10 selectively inhibits the LPS transcriptional response in Mφs. Pathway enrichment analysis indicated an overrepresentation of transcripts associated with interferon and cytokine signaling among the LPS-inducible genes that were significantly repressed by IL-10 in control Mφ (Fig. S5 C). Consistent with this, transcription factor enrichment analysis also showed a significant enrichment of targets of IRF1, IRF2, IRF7, and IRF8 as well as these transcription factors themselves, all of which are involved in Mφ activation and the inflammatory response (Taniguchi et al., 2001; Fig. S5 C). By contrast, genes that were down-regulated by LPS but rescued by IL-10 in control Mφs were overrepresented in pathways related to carbohydrate and lipid metabolism, suggesting alterations in metabolism. Furthermore, these genes were enriched in targets of the transcription factors PPARγ (Ricote et al., 1998), LXR (Joseph et al., 2003), and ATF-3 (Gilchrist et al., 2006; Fig. S5 D), known negative regulators of Mφ activation.

### A subset of IBD GWAS candidate genes are regulated by IL-10 in Mφs

**Figure 5.**
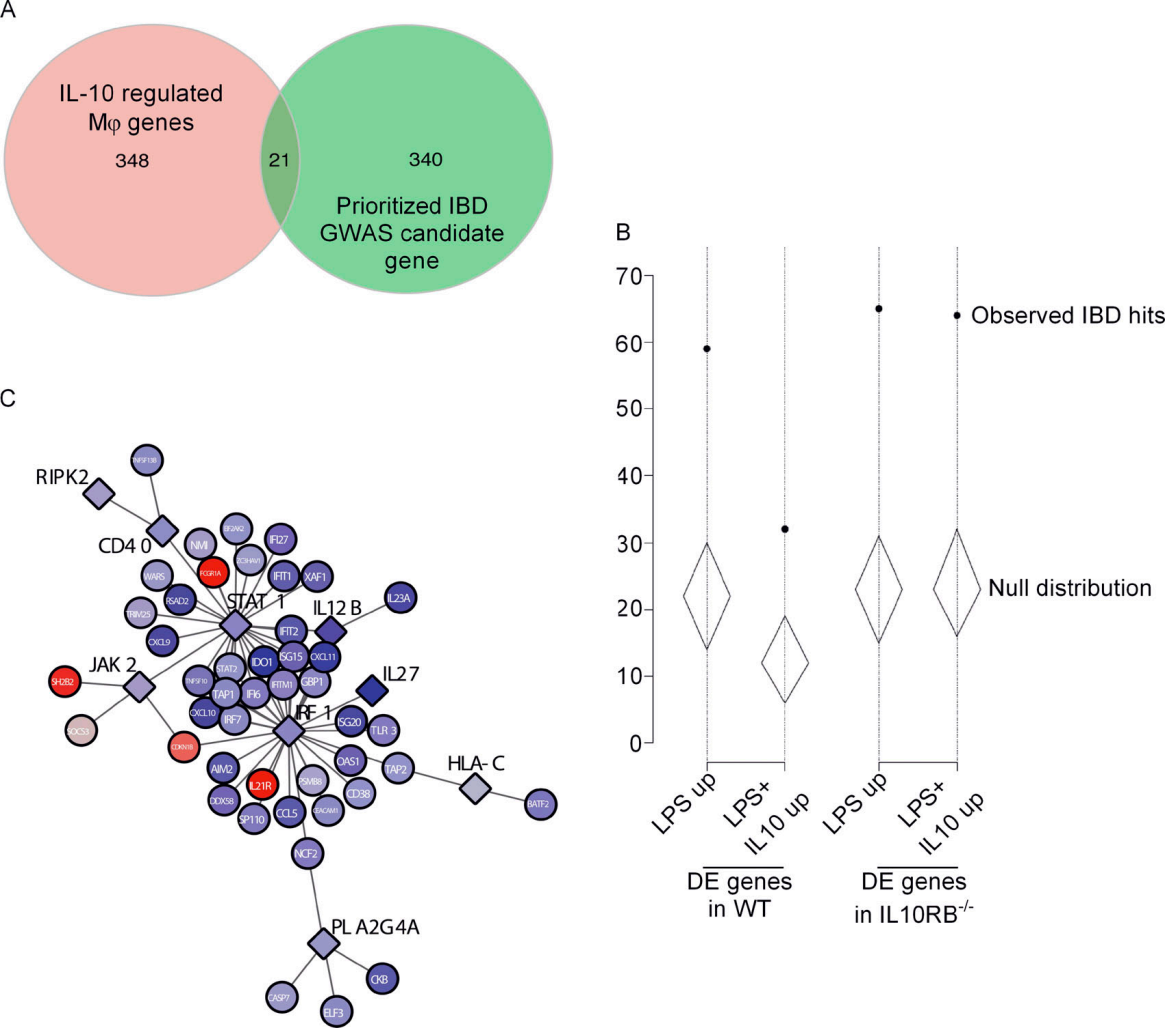
**Overlap between IL-10–regulated genes and IBD GWAS candidate genes. (A)** Venn diagram between IL-10–regulated genes (red; includes genes deregulated by IL-10 treatment alone and IL-10–repressed/rescued genes in LPS-stimulated WT control Mφs but unchanged in IL-10RB^−/−^ Mφs) and all the previously described prioritized gene candidates identified in IBD GWAS (green). **(B)** Number of IBD GWAS risk loci containing genes up-regulated in various treatment conditions (solid black circles), in comparison to null distributions generated from gene lists matched for Mφ expression level and gene length (represented by diamonds showing the region where 95% of null simulations fall). DE, differentially expressed. **(C) **The 21 overlapping genes identified in A were used as input for a network analysis in InnateDB with default settings to identify all the genes that are functionally connected with these 21 (not shown). In this larger network, all genes that are significantly deregulated in control Mφs after combined IL-10 plus LPS stimulation relative to LPS stimulation were identified and represented as a subnetwork using the Cytoscape visualization tool. The red and blue colors represent up- and down-regulation, respectively, of genes after combined IL-10 plus LPS treatment compared with LPS stimulation alone. The increase in intensity for each color represents relative levels of up- or down-regulation compared with LPS stimulation alone. The input IBD candidate genes are shown as diamonds, and their network connections are shown as circles.

Since IL-10 deficiency leads to intestinal inflammation driven by activated Mφs, we set out to identify the IL-10–regulated functional gene network that becomes dysregulated in IL-10 deficiency. Consequently, we compared the list of prioritized gene candidates identified in IBD GWAS (Jostins et al., 2012; Liu et al., 2015) with a list of 369 IL-10–regulated genes we identified in Mφs. These include: (i) genes that are selectively up- or down-regulated by IL-10 stimulation alone in WT Mφs (Fig. 4 B and Table S3); (ii) LPS-induced genes that are selectively repressed in WT Mφs after IL-10 plus LPS stimulation (Fig. 4 C and Table S4); and (iii) LPS down-regulated genes that are selectively rescued in WT Mφs after IL-10 plus LPS stimulation (Fig. 4 D and Table S5). We found 21 genes in common between 361 IBD GWAS candidates and our 369 IL-10–regulated genes (Fig. 5 A, Table S6, and Table S7). Next, we set out to test whether IBD GWAS candidate genes were significantly enriched among IL-10–repressed genes. We compared all Mφ-expressed genes with prioritized IBD GWAS candidates and calculated how many of these IBD candidates were randomly present (null distribution) among LPS and IL-10 plus LPS conditions in both WT and IL-10RB^−/−^ Mφs, taking into consideration the number of differentially expressed genes in each of these conditions. Next, we calculated the actual enrichment of IBD candidates in the above conditions and plotted them on their respective null distribution (Fig. 5 B). The analysis showed that after LPS stimulation, IBD candidate genes are significantly enriched among LPS-inducible genes in both WT and IL-10RB^−/−^ Mφs. However, the number of up-regulated IBD candidate genes was strikingly reduced in the IL-10 plus LPS treatment conditions in WT Mφs but not in IL-10RB^−/−^ Mφs. Genes that were down-regulated by IL-10 during the LPS response were significantly enriched in IBD GWAS loci (3.26-fold enrichment, P < 1 × 10^−4^), whereas genes that were up-regulated by IL-10 during the LPS response showed no enrichment (0.83-fold, P = 0.709). Thus, these results identify a subset of prioritized IBD GWAS candidates that are significantly enriched among LPS-induced genes in Mφs whose expression is repressed by IL-10 treatment. Next, we identified all the genes that are predicted to be functionally connected with the 21 IL-10–regulated IBD candidate genes (Fig. 5 C) using the InnateDB functional network analysis tool (Breuer et al., 2013). By comparing which of these network connections are also regulated by IL-10 in our Mφ RNASeq analysis, we identified a subnetwork linking IL-10–regulated GWAS candidates and their functional network connections that are also regulated by IL-10 in Mφs (Fig. 5 C). Expression patterns of these subnetwork members in our experimental conditions revealed LPS-inducible genes that were down-regulated in the combined IL-10 plus LPS condition (shown in blue), and LPS-repressed genes that were rescued by combined treatment (shown in red) in WT Mφs but not in IL-10RB^−/−^ Mφs. This analysis showed that a number of the IL-10–regulated IBD GWAS candidate genes and their associated networks are strongly down-regulated in the WT control Mφs after combined treatment compared with LPS treatment alone. These include hub genes such as IRF1 (Kamijo et al., 1994; Langlais et al., 2016), STAT1 (Kovarik et al., 1998), and JAK2 (Kakar et al., 2005; Okugawa et al., 2003), which are well-known inflammatory genes and inducers of Mφ activation. By contrast, only a few targets were up-regulated following IL-10 treatment, which included known negative regulators of Mφ activation such as IL-21R (Fabrizi et al., 2014; Fina et al., 2008; Wang et al., 2016), SOCS3, FCGR1A, and SH2B2. Together, these data identify an IL-10–regulated core functional module associated with IBD GWAS genes, which in the absence of IL-10–mediated control may drive excessive Mφ activation in the context of IBD.

### Genes involved in the PGE2 pathway are overexpressed in IL-10RB^−/−^ Mφs

**Figure 6.**
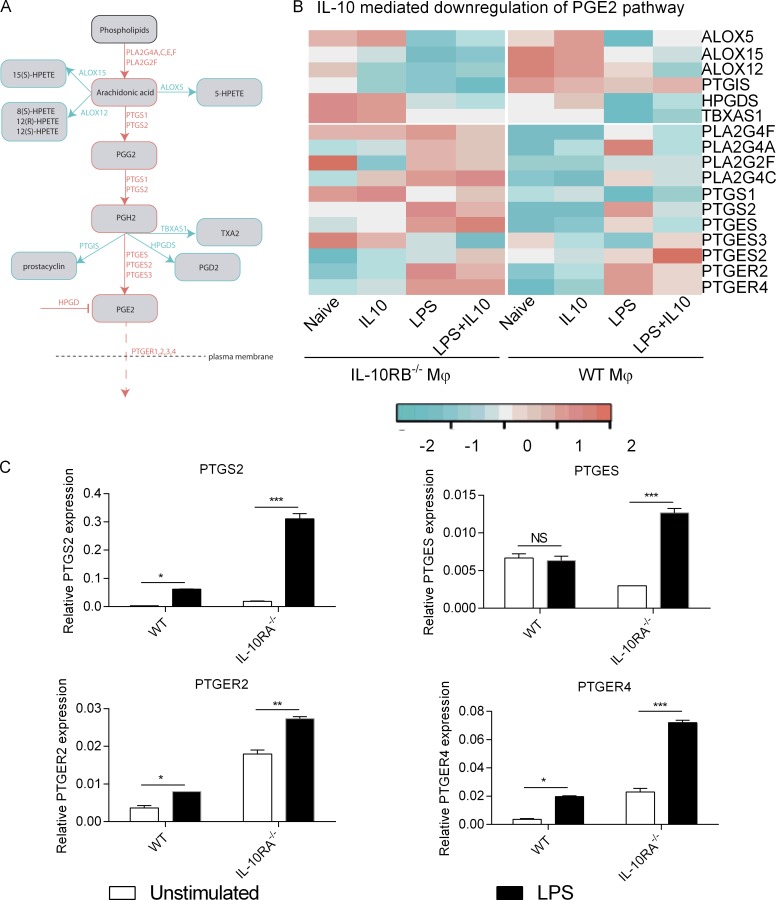
**Loss of IL-10 signaling leads to overexpression of genes involved in the PGE2 pathway. (A)** A simplified schematic diagram of the arachidonic acid pathway from the Kyoto Encyclopedia of Genes and Genomes database showing key steps in the generation of different classes of eicosanoids with a specific focus on the PGE2 synthesis pathway and its receptors. **(B)** Heat map showing average expression values of genes involved in PGE2 synthesis and its receptors (lower panel) and key rate-limiting genes involved in the synthesis of other eicosanoids (upper panel) in IL-10RB^−/−^ (*n* = 3) and control Mφs (*n* = 4) after different treatments. **(C)** Relative mRNA expression for genes involved in PGE2 synthesis (PTGS2 and PTGES) and PGE2 receptors (PTGER2 and PTGER4) between IL-10RA^−/−^ Mφs and isogenic control kolf_2 Mφs with or without LPS stimulation analyzed by RT-qPCR. Data from at least triplicate wells of each condition are presented as means ± SD and are representative of at least three independent experiments. Two-way ANOVA with Tukey’s multiple comparisons test was used to assess statistical significance. *, P < 0.05; **, P < 0.01; ***, P < 0.001.

The IL-10–regulated IBD GWAS functional network included a phospholipase A2 member PLA2G4A (Fig. 5 C). Phospholipases catalyze the first rate-limiting step in the synthesis of arachidonic acid–derived eicosanoid lipid mediators including PGE2 (Fig. 6 A). In addition to PLA2G4A, several other genes in the eicosanoid pathway such as PTGS2 and PTGER4 have been identified as potential susceptibility candidates in IBD GWAS, suggesting a potential role for the PGE2 pathway in intestinal homeostasis (Liu et al., 2015). We compared the expression patterns of key genes involved in PGE2 synthesis as well as PGE2 receptor genes between WT and IL-10RB^−/−^ Mφs using our transcriptomic data (Fig. 6 B). Only those genes that are selectively involved in PGE2 biosynthesis and its receptor pathways were overexpressed in IL-10RB^−/−^ compared with control Mφs, especially after LPS stimulation (Fig. 6 B, bottom). Strikingly, genes that are involved in the synthesis of other eicosanoid species were not different between control and IL-10RB^−/−^ Mφs (Fig. 6 B, top). To independently validate this data, IL-10RA^−/−^ and its isogenic control kolf_2 Mφs were stimulated with LPS as before, and expression levels of PTGS1, PTGS2, PGES, PTGER2, and PTGER4 mRNA were compared by RT-qPCR. Expression of these PGE2 pathway genes was more robustly induced in IL-10RA^−/−^ Mφs compared with WT controls (Fig. 6 C). Taken together, these findings suggest that in the absence of IL-10 signaling, genes associated with the PGE2 pathway are more robustly induced in Mφs after LPS stimulation.

### IL-10RB^−/−^ Mφs produce higher amounts of PGE2 that limits their antimicrobial capacity

**Figure 7.**
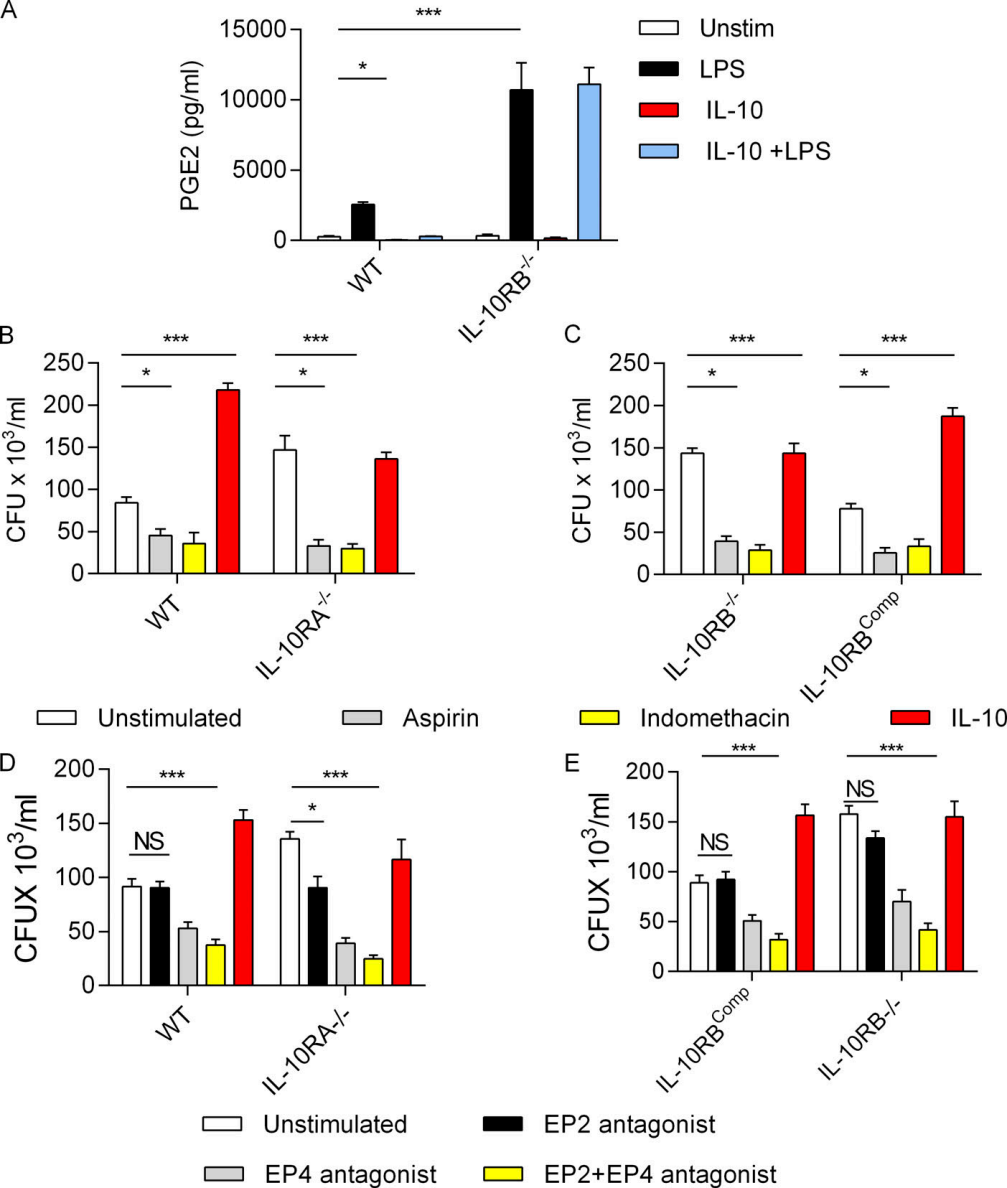
**Excessive PGE2 production limits antimicrobial capacity of IL-10RB**^**−/−**^** Mφs. (A)** PGE2 levels measured by ELISA in supernatants from IL-10RB^−/−^ and control kolf_2 Mφs (*n* = 4) prestimulated overnight with 20 ng/ml rhIL-10 or unstimulated (Unstim) and further stimulated with 2 ng/ml LPS for 6 h in the presence or absence of IL-10 as appropriate. **(B and C)** Bacterial survival measured at a 5-h time point by gentamicin protection assay and reported as CFU × 10^3^/ml in IL-10RA^−/−^ Mφs (*n* = 4) compared with isogenic control kolf_2 Mφs (*n* = 4; B) and in IL-10RB^comp^ Mφs (*n* = 4) compared with IL-10RB^−/−^ Mφs (*n* = 4; C) prestimulated for 2 h with COX2 inhibitors aspirin (10 µM) or indomethacin (10 µM) or left unstimulated and then infected with 10 MOI of *S.* Typhimurium SL1344 (pssaG:GFP) in the presence or absence of COX2 inhibitors. **(D and E)** Bacterial survival as in B and C in IL-10RA^−/−^ Mφs (*n* = 4) compared with isogenic control kolf_2 Mφs (*n* = 4; D) and in IL-10RB^comp^ Mφs (*n* = 4) compared with IL-10RB^−/−^ Mφs (*n* = 4; E) stimulated with 20 nM EP2, EP4, and EP2 plus EP4 antagonists or left unstimulated. In these experiments, inhibitors were added after initial bacterial uptake. Data shown in all panels are from at least quadruplicate wells, are presented as means ± SD, and are representative of at least three independent experiments. Two-way ANOVA with Tukey’s multiple comparisons test was performed using GraphPad software to assess statistical significance. ***, P < 0.001; *, P < 0.05.

Next, we directly measured PGE2 levels in cell culture supernatants. IL-10RB^−/−^ Mφs secreted significantly higher amounts of PGE2 compared with control Mφs after LPS stimulation (Fig. 7 A), consistent with a previous report that Mφs from myeloid-specific IL10RA^−/−^ mice produced higher amounts of PGE2 (Zigmond et al., 2014). Furthermore, IL-10 inhibited LPS-induced PGE2 secretion in control but not in IL-10RB^−/−^ Mφs (Fig. 7 A), confirming that in the absence of IL-10 signaling, PGE2 production is enhanced. Since previous studies suggested PGE2 can compromise the microbicidal capacity of Mφs (Canetti et al., 2007; Goldmann et al., 2010; Mittal et al., 2010; O’Brien et al., 2014; Rogers et al., 2014; Serezani et al., 2007; Serezani et al., 2012), we tested whether the reduced microbicidal capacity in IL-10RB^−/−^ Mφs is a consequence of overproduction of PGE2. To this end, we compared the amount of intracellular *S*. Typhimurium killing in WT, IL-10RA^−/−^, IL-10RB^−/−^, and IL-10RB^comp^ Mφs after pretreatment with two different pharmacological inhibitors of PGE2 synthesis, aspirin and indomethacin. We show that both aspirin and indomethacin treatment significantly improved bacterial killing in all Mφ populations (Fig. 7, B and C). However, aspirin and indomethacin are not selective inhibitors of PGE2 synthesis; rather, they block all eicosanoids by inhibiting PTGS2. Therefore, to show a specific effect of PGE2 on bacterial killing, we also compared the intracellular killing of *S*. Typhimurium in WT and IL-10RA^−/−^ Mφs as well as IL-10RB^−/−^ and IL-10RB^comp^ Mφs in the presence of selective inhibitors of PTGER2 (PF-04418948) and PTGER4 (L-161,982) either individually or in combination (Fig. 7, D and E). We show that PTGER2 antagonism had only a modest effect on bacterial killing, whereas PTGER4 or combined PTGER2 and PTGER4 inhibition showed the most striking increase in bacterial killing, indicating a more dominant role of PTGER4 in suppression of bacterial killing, an effect which was most prominent in IL-10RA^−/−^ and IL-10RB^−/−^ Mφs compared with control Mφs (Fig. 7, D and E). Taken together, we conclude that in the absence of IL-10 signaling, PGE2 synthesis is enhanced in IL-10RB^−/−^ and IL-10RA^−/−^ Mφs, which in turn reduces their microbicidal capacity.

## Discussion

Here, we report a reciprocal regulatory axis between the IL-10 and PGE2 pathways as a key regulator of Mφ activation and intestinal homeostasis. A delicate balance between these two pathways is critical for optimal host defense in the absence of excessive Mφ activation and tissue damage. We show that loss of IL-10 signaling induces a microbial hyper-responsiveness and overproduction of PGE2 in Mφs, which in turn limits their microbicidal capacity. This toxic combination of hyperactive Mφs and reduced bacterial clearance may fuel chronic intestinal inflammation as a consequence of IL-10/IL-10R deficiencies.

Mφs differentiated from mutant patient-derived iPSCs recapitulate the functional defects of primary Mφs (Flynn et al., 2015; Panicker et al., 2012). The hyperinflammatory and IL-10–unresponsive phenotype observed in IL-10RB^−/−^ iPSC-derived Mφ is consistent with similar functional assays performed using primary leukocytes from that patient (Engelhardt et al., 2013). Thus, patient-derived and genome-engineered iPSCs can be used to model specific aspects of human IBD.

The IL-10RB^−/−^ iPSCs successfully differentiated into Mφs, suggesting that IL-10 signaling is not critical for iPSC-to-Mφ differentiation, consistent with previous findings (Lang et al., 2002a). IL-10RA^−/−^ mice develop intestinal pathology and systemic immune activation at weaning (Redhu et al., 2017), and bone marrow–derived Mφs from those mice are reported to be skewed toward a “M1-like” activation state even without stimulation (Shouval et al., 2014a), possibly reflecting inflammatory conditioning in vivo. We did not detect an overt M1 phenotype in IL-10RB^−/−^ Mφs, most likely because these cells have not been exposed to inflammatory preconditioning.

How IL-10 exerts its anti-inflammatory response is not fully elucidated; our transcriptomic analysis provided some potential clues that warrant further investigation. IL-10 stimulation alone affects the expression of only a few genes in control Mφs, including SOCS3 (Croker et al., 2003; Wong et al., 2006) and MARCH1 (Galbas et al., 2017; Hunt et al., 2012; Mittal et al., 2015) with known anti-inflammatory and Mφ deactivation functions. However, consistent with previous findings, the IL-10 effect was most prominent in repression of LPS-induced inflammatory genes (Gopinathan et al., 2012; Lang et al., 2002a; Murray, 2005), possibly through epigenetic mechanisms (Conaway et al., 2017; Kobayashi et al., 2012; Simon et al., 2016). In addition, IL-10 also induced expression of LPS-repressed genes including those related to carbohydrate and lipid metabolism, in line with a recent report that the anti-inflammatory effects of IL-10 involve metabolic reprogramming of Mφs (Ip et al., 2017). IL-10 also rescued LPS-induced suppression of transcription factors PPARg (Ricote et al., 1998), LXR (Joseph et al., 2003), and ATF-3 (Gilchrist et al., 2006), all of which are involved in regulating the Mφ inflammatory response.

An intriguing new finding is the enrichment of IBD GWAS candidates among IL-10-repressed LPS-inducible genes. Among these are a number of cytokines and transcriptions factors centered on the IL-12/IL-23 axis and interferon response genes, several of which are known to mediate intestinal inflammation (Maloy and Powrie, 2011). Thus, this network of genes that includes known inflammatory transcription factors JAK2, STAT1, and IRF1 as hub genes and their transcriptional targets represents an IL-10–regulated functional module that when dysregulated in IL-10R–deficient settings may lead to pathogenic Mφ activation and intestinal inflammation. Enrichment of IBD GWAS candidates raises the possibility that alterations in this functional module may be a biomarker and therapeutic target of deranged Mφ activation in polygenic IBD.

Perhaps the most significant finding of our study is that IL-10RB^−/−^ and IL-10RA^−/−^ Mφs showed a marked deficiency in bacterial killing. This is arguably counterintuitive, as IL-10 treatment also reduced bacterial killing in WT Mφs (Fig. 3) as described by others (Fleming et al., 1999; Lang et al., 2002b; Lee et al., 2011; Oswald et al., 1992). Thus, IL-10 treatment and IL-10 blockade both compromise the microbicidal capacity of Mφs. We present evidence that the reduced antibacterial properties of IL-10R–deficient Mφs is due to enhanced PGE2 secretion. Previous studies have highlighted a negative feedback loop between IL-10 and PGE2. Thus, PGE2 induced in response to innate immune stimulation augments IL-10 secretion and signaling (Alvarez et al., 2009; Cheon et al., 2006; Harizi et al., 2002), which in turn limits PGE2 secretion in an autocrine fashion (Berg et al., 2001; MacKenzie et al., 2013; Niiro et al., 1997; Niiro et al., 1994). Hence, in the absence of IL-10R signaling, the negative regulatory arm of this feedback loop is broken, leading to enhanced secretion of PGE2. Thus, the enhanced IL-10 secretion observed in IL-10RB^−/−^ Mφs (Fig. S2) could be driven by enhanced PGE2 signaling in these cells. PGE2 excretion is raised in the stools of patients with ulcerative colitis (Gould, 1975). In untreated patients with ulcerative colitis in remission and in relapse, mucosal PGE2 release was highly increased compared with controls (Rampton et al., 1980). Human genetic studies have also identified a number of genes in the PGE2 pathway as susceptibility candidates for IBD (Libioulle et al., 2007; Liu et al., 2015). PGE2 blockade by nonsteroidal anti-inflammatory drugs (Lanas, 2009) and monogenic loss of function mutations in PLA2G lead to early onset and severe intestinal pathologies mimicking the pathogenic side effects of nonsteroidal anti-inflammatory drugs (Brooke et al., 2014; Maddirevula et al., 2016). Similarly, in a murine model, monocyte-derived PGE2 is essential for protection against immunopathology during acute intestinal infection (Grainger et al., 2013), suggesting that the PGE2 pathway promotes intestinal homeostasis.

However, the role of PGE2 in inflammation is complex (Harris et al., 2002). It is generally considered as an inflammatory mediator primarily due to its effect on the vasculature. However, it also has significant immunoregulatory effects, especially on myeloid cells by limiting their inflammatory and host defense response (Agard et al., 2013; Medeiros et al., 2012). There are four distinct G protein coupled receptors (EP1-4) for PGE2. They have distinct tissue distribution, binding affinities, and signaling specificities that together determine their pro- or anti-inflamatory functions (Rodríguez et al., 2014). Mφs predominantly express EP2 and EP4, which limit their host defense function by inhibiting TLR signaling (Perkins et al., 2018; Strassmann et al., 1994). PGE2 also influences phagocytic uptake, oxidative burst, and autophagic killing of cytosolic bacteria (Canetti et al., 2007; Goldmann et al., 2010; Martínez-Colón et al., 2018; Mittal et al., 2010; O’Brien et al., 2014; Rogers et al., 2014; Serezani et al., 2007; Serezani et al., 2012). In addition, PGE2 also affects Mφ metabolism and associated Mφ polarization, mitochondria membrane potential, and endoplasmic reticulum function (Sanin et al., 2018); these in turn can influence a multitude of host defense mechanisms in Mφs. Many of these are shown to be particularly important for defense against *S*. Typhimurium (Thomas et al., 2017; West et al., 2011). Thus, we envisage that PGE2 overproduction may have impacted many of these microbicidal mechanisms, resulting in reduced bacterial killing in IL-10RA^−/−^ and IL-10RB^−/−^ Mφs. In addition, pharmacological blockade of PGE2 synthesis and its receptors rescued this phenotype in IL-10RB^−/−^ and IL-10RA^−/−^ Mφs, further highlighting the pathogenic relevance of the PGE2-mediated aberrant antimicrobial response in IL-10 signaling deficiencies. Selective inhibition of EP2 and EP4 revealed their differential influence on antibacterial mechanisms, with EP4 being more dominant. This is an intriguing new observation with potential therapeutic implications and warrants further investigation using genetic approaches to establish the relative impact of these two receptors on Mφ antimicrobial properties.

IL-10 and PGE2 induce overlapping yet distinct inhibitory effects on Mφs (Brencicova et al., 2017). Further studies are required to decipher the relative anti-inflammatory functions of IL-10 and PGE2 in the intestine. However, clinical studies have shown that the PGE2 pathway is strongly induced in both pediatric and adult IBD patients, and PGE2 metabolites are reliable biomarkers of intestinal inflammation (Arai et al., 2014; Hagiwara et al., 2017), indicating a general involvement of this pathway in common forms of IBD beyond rare diseases such as IL-10 deficiency.

In summary, independent clinical and genetic evidence implicates IL-10 and PGE2 pathways in the maintenance of intestinal homeostasis. Here, we connect these two pathways mechanistically, suggesting that they function as an integrated regulatory module to control Mφ function and that dysregulation of this balance in the intestine may have important consequences for both host defense against infection and maintenance of intestinal homeostasis.

## Materials and methods

### Patient with IL-10RB mutation

The phenotype and underlying genetic mutation of this infantile-onset IBD patient have been reported (Engelhardt et al., 2013). Briefly, the female patient of Arabic origin was born to a consanguineous marriage. Two siblings died in the first few months of life due to severe intestinal inflammatory disease. At the age of 6 mo, the patient developed diarrhea, fever, and perianal disease. She was treated with steroids. At the age of 9 mo, a subtotal colectomy with an ileostomy was performed, but perianal disease persisted. Steroids were finally stopped at the age of 9 yr, but perianal disease persisted. Diverse treatments with infliximab and methotrexate, certolizumab, and adalimumab controlled neither the colitis nor the perianal disease. The patient experienced multiple extraintestinal manifestations including finger sepsis, poor dentition, gum hyperplasia, chronic paronychia, ulcerated perineum, and hypertrophic skin. At the age of 12 yr, an EBV lymphoma was diagnosed, and chemotherapy was initiated. Genetic analyses revealed a homozygous splice site mutation in *IL10RB* gene at nucleotide IVS3G>C, resulting in exon 3 skipping, frame shift, premature stop codon, and a predicted truncated IL-10RB protein at amino acid position 72 (p.Leu59fsX72) that is consistent with the phenotype of the patient (Engelhardt et al., 2013). Most recently, she developed a new non-Hodgkin’s lymphoma of sclerosing type.

### Human experimental guidelines approval statement

A favorable ethical opinion was granted by the National Research Ethics Service Research Ethics Committee Yorkshire and The Humber—Leeds West (UK), reference number 15/YH/0391.

### Generation of iPSCs from IL-10RB^−/−^ patient fibroblasts

#### Reprogramming

A skin biopsy was taken from the patient to generate fibroblasts. 5 × 10^5^ cells were plated in one well of a 6-well plate in fibroblast growth medium. The next day, the cells were transduced with Sendai viruses encoding the transcription factors hOCT4, hSOX2, hKLF4, and hc-MYC using a multiplicity of infection (MOI) of 3 and then incubated overnight at 37°C in a 5% CO_2_ incubator. The virus-containing medium was replaced on the next day with fresh fibroblast growth medium and cultured further for 4 d in the same medium. Starting from day 5 after transduction, cells were maintained in iPSC media (knockout serum replacement plus fibroblast growth factor 2 [FGF2]) and changed daily. 10–21 d after transduction, the transduced cells began to form colonies with iPSC morphology, and visible colonies were handpicked and transferred onto 12-well inactivated MEF feeder plates. Colonies were expanded into 6-well MEF-coated feeder plates and passaged every 5–7 d (depending on the confluence of the plates) at a desired ratio.

#### Culture and propagation

To expand colonies, iPSCs were grown on irradiated MEF feeders (Globalstem) using the stem cell media described below. Advanced DMEM (Life Technologies) was supplemented as follows: 10% Knockout Serum Replacement (Life Technologies), 2 mM L-glutamine (Life Technologies), 0.14% 2-mercaptoethanol (Sigma-Aldrich), and 4 ng/µl of recombinant human basic FGF2. Media were changed daily, and the cells were passaged every 7 d depending on the confluence of the plates. Fibroblasts were grown in media consisting of Advanced DMEM, 10% FBS, 1% pen/strep, 1% L-glutamine, and 0.07% 2-mercaptoethanol.

#### Passaging

To passage iPCS, cells were washed with PBS and incubated with collagenase (Collagenase IV, 1 mg/ml, 17104–019; Invitrogen) and dispase (1 mg/ml, 17105–041; Invitrogen) for 45 min. After that, colonies were collected in a falcon tube containing iPSC media and were allowed to sediment for 2 min. The supernatant, containing residual collagenase/dispase, was removed, and the colonies were washed once with iPSC medium. The colonies were allowed to sediment again, and the supernatant was removed. Finally, colonies were mechanically broken up and plated onto fresh MEF feeders. Cells were passaged every 5–7 d (depending on the confluence of the plates) at a desired ratio.

### Differentiation of IL-10RB^−/−^ iPSCs into three germ layers

The IL-10RB^−/−^ iPSCs were characterized by assessing their pluripotency state as well as their ability to differentiate into three germ layers using methods described previously (Agu et al., 2015). Briefly, for pluripotency assays, colonies were grown on MEF feeder plates, and expression levels of pluripotency markers OCT4, SOX-2, and NANOG were assessed by immunohistochemistry. For differentiation into mesoderm, endoderm, and neuroectoderm, cells were cultured overnight in predifferentiation medium CDM-PVA (chemically defined medium with poly venyl alcohol) supplemented with recombinant Activin-A (10 ng/ml; Cambridge Stem Cell Institute [CSCR], University of Cambridge) and zebrafish FGF2 (12 ng/ml; CSCR, University of Cambridge). For differentiation into mesoderm following culture in predifferentiation media, spent media were removed and replaced with fresh CDM-PVA medium containing bone morphogenic protein 4 (BMP4, 10 ng/ml; R&D Systems), FGF2 (20 ng/ml; CSCR, University of Cambridge), recombinant Activin-A (10 ng/ml; CSCR, University of Cambridge), LY29004 (10 mM; Promega), and CHIR99021 (5 mM; Selleck Chem) and subsequently cultured for 3 d with daily media change. Expression of mesodermal marker Bracyury, Mixl1, and Eomes were determined by immunohistochemistry. For differentiation into endoderm, following culture in predifferentiation media, cells were cultured further in differentiation media for 3 d. Briefly, day 1 spent media was removed and replaced with fresh CDM-PVA medium supplemented with recombinant Activin-A (100 ng/ml; CSCR, University of Cambridge), zebrafish FGF2 (80 ng/ml; CSCR, University of Cambridge), BMP4 (10 ng/ml; R&D Systems), LY29004 (10 mM), and CHIR99021 (3 mM). Day 2 media were removed and replaced with fresh CDM-PVA medium supplemented with recombinant Activin-A (100 ng/ml), zebrafish FGF2 (80 ng/ml), BMP4 (10 ng/ml), and LY29004 (10 mM). Day 3 media was removed and replaced with RPMI medium supplemented with B27 (13; Life Technologies), recombinant Activin-A (100 ng/ml), zebrafish FGF2 (80 ng/ml), and Non-Essential Amino Acids (13; Life Technologies) with daily media change. Expression of endodermal marker GATA4 was determined by immunohistochemistry. For differentiation to neuroectoderm, iPSCs were grown for 12 d in CDM-PVA medium supplemented with SB431542 (10 mM; Tocris Bioscience), FGF2 (12 ng/ml; CSCR, University of Cambridge), and NOGGIN (150 ng/ml; R&D Systems) with daily media change. Expression of neuroectoderm marker Nestin1 was determined by immunohistochemistry.

### Genome editing and overexpression experiments

Detailed methods for generating IL-10RA^−/−^ iPSCs by CRISPR/CAS9-based genome editing in the control kolf_2 line (Yeung et al., 2017) and complementation of IL-10RB^−/−^ iPSCs by introducing functional copies of IL-10RB gene using a TALEN-based approach have been previously reported by our group (Forbester et al., 2018).

### Generation of IL-10RA^−/−^ IPSCs by CRISPR technology

Control kolf_2 cells were adapted to feeder-free culture, and several sublines were isolated by single-cell cloning. The subline kolf_2-C1 was used for this study, and these cells showed a stable, normal karyotype (46; XY) for ≤25 passages. Biallelic knockouts in kolf_2 human iPSCs were generated using a method that was found to minimize the potential for off-target effects. Briefly, the intermediate targeting vector for each gene was generated by Gibson assembly of the four fragments: puc19 vector, 5′ homology arm, R1-*pheS/zeo*-R2 cassette, and 3′ homology arm. The homology arms were amplified by PCR from kolf_2 human iPSC genomic DNA. pUC19 vector and R1-*pheS/zeo*-R2 cassette were prepared as gel-purified blunt fragments (EcoRV digested), while the PCR fragments were either gel purified or column purified (QIAquick; Qiagen). The resultant Gibson assembly reactions (Gibson Assembly Master Mix; NEB) were transformed into NEB 5-alpha competent cells, and clones resistant to carbenicillin (50 µg ml^−1^) and zeocin (10 µg ml^−1^) were analyzed by Sanger sequencing to verify all junctions. Subsequently, the intermediate targeting vectors were turned into donor plasmids via a Gateway exchange reaction. LR Clonase II Plus enzyme mix (Invitrogen) was used as described previously (Tate and Skarnes, 2011), with the difference that it was a two-way reaction exchanging only the R1-*pheSzeo*-R2 cassette with the pL1-EF1αPuro-L2 cassette. The latter had been generated by cloning synthetic DNA fragments of the EF1α promoter and puromycin into one of the pL1/L2 vectors as described previously (Tate and Skarnes, 2011). Following Gateway reaction and selection on YEG + carbenicillin (50 µg ml^−1^) agar plates, correct donor plasmids were confirmed by Sanger sequencing of all junctions. Plasmids carrying single guide RNA sequences were generated by cloning forward and reverse strand oligos into the BsaI site of either U6_BsaI_gRNA or p1260_T7_BsaI_gRNA vectors (kindly provided by Sebastian Gerety, Wellcome Trust Sanger Institute, Cambridge, UK). Kanamycin-resistant clones (50 µg ml^−1^) were isolated, and cloning of the correct sequence was verified by Sanger sequencing.

Human iPSCs were dissociated to single cells and nucleofected (Amaxa2b nucleofector; Lonza) with Cas9 coding plasmid (hCas9, 41815; Addgene), single guide RNA plasmid, and donor plasmid. Following nucleofection, cells were selected for ≤11 d with 0.25 µg ml^−1^ puromycin. Individual colonies were picked into 96-well plates, grown to confluence, and then replica plated. Once confluent, the replica plates were frozen as single cells in 96-well vials, or the wells were lysed for genotyping.

To genotype individual clones from 96-well replica plates, cells were lysed and used for PCR amplification with LongAmp Taq DNA Polymerase (NEB). Insertion of the cassette into the correct locus was confirmed by visualizing on 1% E-gel (Life Technologies) PCR products generated by gene-specific (GF1 and GR1) and cassette-specific (ER and PF) primers for both 5′ and 3′ ends. We also confirmed single integration of the cassette by performing an RT-qPCR copy number assay. To check the CRISPR site on the nontargeted allele, PCR products were generated either from across the locus, using the 5′ and the 3′ gene-specific genotyping primers (GF1-GR1), or from around the site using primers 5F-3R that would amplify a short, ∼500-bp, amplicon. In both cases, the PCR products were treated with exonuclease and alkaline phosphatase (NEB) and Sanger sequenced using primers SF and SR.

### Complementation of IL-10RB^−/−^ iPSCs

To restore expression of functional *IL10RB* gene in the IL-10RB^−/−^ patient iPSCs, we used the TALEN-mediated gene integration approach to integrate a functional copy of the *IL10RB* gene into the genome of the IL-10RB^−/−^ mutant patient iPSCs. In brief, we generated the AAVS1 EF1a-IL10RB-PGK-puro targeting vector by Gibson assembly. We transformed the Gibson assembly product into OneShot TOP10 chemically competent *Escherichia coli* (Thermo Fisher Scientific) and picked positive colonies. We isolated plasmids from the positive colonies and confirmed the presence and sequence of EF1α-*IL10RB* in the targeting vector by restriction digests, PCR and sequencing. Subsequently, the targeting vector was transformed into competent *E. coli* to isolate endotoxin-free plasmids to transform into the IL-10RB^−/−^ patient iPSCs. We transfected the mutant human iPSCs with TALEN-L (5′-CCC​CTC​CAC​CCC​ACA​GT-3′), TALEN-R (5′-TTT​CTG​TCA​CCA​ATC​CT-3′) and targeting vector via nucleofection (Amaxa Biosystems). The resultant targeted cells were selected on puromycin for 7 d. Surviving colonies were picked and expanded. The positive clones were confirmed by PCR and sequencing. All iPSC lines used in this study are karyotypically normal.

### Directed differentiation of iPSCs into mature Mφs

The detailed protocol for iPSC-to-Mφ differentiation has been described (Alasoo et al., 2015; Hale et al., 2015). Briefly, for Mφ differentiation, iPSC colonies grown on mouse feeders were detached with collagenase and dispase and transferred into low adherent bacteriological plastic and cultured for another 3 d using iPSC base media without FGF2 to form a three-germ layer containing embryoid bodies. After 3 d, for long-term production of myeloid precursors, embryoid bodies were transferred into gelatin-coated tissue culture plastic dishes in X-vivo 15 medium supplemented with 25 ng/ml IL-3 and 50 ng/ml M-CSF (both R&D Systems). Terminal differentiation of myeloid precursors into mature Mφs was achieved by culturing myeloid precursors in high concentrations of M-CSF (100 ng/ml) in RPMI medium supplemented with 10% FCS, L-glutamine, penicillin, and streptomycin.

### Bacteria and growth conditions

*S.* Typhimurium SL1344 harboring the reporter plasmid pssaG::GFP was grown on L-broth or L-agar containing ampicillin at 100-μg/ml final concentration. *S*. Typhimurium SL1344(pssaG::GFP) has been described previously (McKelvie et al., 2004). Briefly, the promoter region of *ssaG* was cloned into plasmid pQF50, upstream of a promoterless GFP gene derived from pmutGFP3.1 (Promega Laboratories). Growth in conditions favoring the activation of Salmonella Pathogenicity Island-2, of which pssaG is a component that leads to expression of GFP via the *ssaG* promoter region. For the infection studies, we grew the cultures statically overnight to simulate microaerophilic conditions at 37°C. The culture OD at 600 nm was measured, and the CFU/ml was calculated.

### Gentamicin protection assay for assessing bacterial survival within Mφs

Bacterial uptake, intracellular survival, and replication were assessed using a gentamicin protection assay as described previously (Weinstein et al., 1998), with minor modifications. Briefly, 2 × 10^5^ Mφs were plated on 24-well plates in RPMI supplemented with 10% heat-inactivated FCS and 2 mM L-glutamine without antibiotics. As indicated, in some experiments, cells were pretreated overnight with either 20 ng/ml recombinant human IL-10 (rhIL-10) or 20 µg/ml blocking antibodies against human IL-10 (clone 25209), IL-10RA (clone 37607), or IL-10RB (clone 90220). Antibodies were from R&D Systems and added for overnight and kept throughout the killing assay. In some experiments, cells were pretreated for 2 h with either 10 µM aspirin or indomethacin (two well-known COX2 inhibitors). In some other experiments, we used 20 nM PTGER2 antagonist (PF-04418948) and PTGER4 antagonist (L-161,982), either individually or in combination. In these experiments, antagonists were added after initial bacterial uptake to avoid any potential effect on bacterial uptake. Subsequently, the indicated MOI of *S*. Typhimurium was added to the medium containing no antibiotics and incubated at 37°C for 0.5 h to allow bacterial uptake. Cells were washed three times with PBS and incubated for an additional 1 h in medium containing 50 µg/ml gentamicin to kill extracellular bacteria. After incubation, medium was removed and replaced with medium without any antibiotics. After the indicated time points, cells were lysed in 0.1% Triton X-100 in PBS solution, and multiple 10-fold serial dilutions were plated on Luria broth agar containing 100 µg/ml ampicillin. The numbers of intracellular bacteria were determined by counting colonies the next day.

### IL-10 suppression assay and cytokine analysis

Briefly, 1 × 10^5^ Mφs were plated on 96-well plates in RPMI supplemented with 10% FCS and L-glutamine without antibiotics. Some wells were pretreated overnight with 20 ng/ml rhIL-10. The next day, cells were stimulated with 2.5 ng/ml LPS for 6 h in the presence or absence of IL-10. After incubation, supernatants were harvested and stored at −80°C for cytokine analysis. In some experiments, IL-10–pretreated Mφs were infected with *S*. Typhimurium (MOI 1) in the presence or absence of IL-10 as before; after 1 h of incubation, cells were washed three times with PBS to remove extracellular bacteria, and fresh medium was added with or without IL-10 as appropriate. After indicated time points, 25 µl of medium was harvested and stored at −80°C for cytokine analysis and replaced with the same volume of medium for subsequent time points. Cytokine concentrations in LPS-stimulated samples were analyzed by ELISA, and cytokines from *S*. Typhimurium–infected supernatants were analyzed by Luminex assay.

### Measurement of PGE2

The PGE2 level in the tissue culture medium was determined using a commercially available competitive ELISA kit (Abcam) according to the manufacturer’s instructions. The ELISA-based PGE2 measurement is a semiquantitative method that may overestimate absolute levels of PGE2, but accurately reflect the relative abundance of PGE2 between different conditions such as WT and IL-10B^−/−^ Mφs in the context of this study.

### Flow cytometry

The IL-10RB^−/−^ and control Mφs were grown in tissue culture plastic dishes in RPMI medium. Cells were detached using lidocaine-EDTA solution as described previously (Mukhopadhyay et al., 2006). Mφs were transferred into 96-well round-bottom plates at a density of 10^5^ cells/well and incubated for 30 min at 4°C in 100 µl of FACS blocking buffer containing 5% FCS in PBS, 0.1% sodium azide, and 2 µl of Trustain Fc receptor block. After incubation, 5 µl of directly conjugated anti-human antibodies against individual Mφ plasma membrane antigens CD14 A488, CD16 APC-Cy7 (AbD Serotec), and CD206 APC (Becton Dickinson) or appropriate isotype-matched control antibodies with the same fluorophore were added to each well and incubated for an additional 30 min. Cells were washed twice with FACs buffer, resuspended in PBS, and analyzed on a Becton Dickinson FACsAria11 using FACS Diva software. For analysis of intracellular phosphorylated STAT3, cells were cultured for 15 min with 20 ng/ml rhIL-10 fixed with BD cytofix and permeabilized with ice-cold BD perm buffer III stained with anti-pSTAT3 (pY705)-Alexa Fluor 647 (clone 4/P-STAT3) according to the manufacturer’s Phosflow protocol (BD Biosciences). Signals were acquired on a BD LSR Fortessa (BD Biosciences) with FACS-Diva software (BD Biosciences). Data were analyzed using Flowjo v10.1 software.

### RT-qPCR

RNA was isolated from either iPSC-derived Mφs or from primary monocyte-derived Mφs with a Qiagen RNAeasy kit and reverse transcribed with the QuantiTect RT kit (Qiagen) according to the manufacturer’s protocol. All RT-qPCR experiments were performed with TaqMan gene expression assays and TaqMan gene expression master mix (Applied Biosystems) on the Applied Biosystems StepOne real-time PCR system. RT-qPCR data were analyzed via the comparative C_T_ method with GAPDH as an endogenous control. The following TaqMan probes were used for the indicated genes: Hs00168754_m1 (PTGER2), Hs00168761_m1 (PTGER4), Hs00153133_m1 (PTGS2), Hs00610420_m1 (PTGES), and Hs99999905_m1 (GAPDH).

### RNASeq experiment and analysis

RNA was extracted with an RNeasy Mini Kit (Qiagen) according to the manufacturer’s protocol. Standard Illumina poly-A enriched libraries were prepared and then sequenced 5-plex on Illumina HiSeq 2500, generating 20–50 million 75-bp paired-end reads per sample. Sequencing reads were aligned to the GRCh38 reference genome with Ensembl 74 annotations using STAR. Reads overlapping gene annotations were counted using featureCounts (Liao et al., 2014), and DESeq2 (Love et al., 2014) was used to identify differentially expressed genes. Genes with false discovery rate (FDR) <0.05 and fold-change >2 were identified as differentially expressed. All downstream analysis was performed in R (R Development Core Team, 2008), and ggplot2 (Wickham, 2009) and the base package were used for figures. Differential expression was assessed using the DESeq function to retrieve the result tables and filtered according to the cutoffs as above. Differences in the response of the WT Mφs to different conditions compared with the IL-10RB^−/−^ Mφs were assessed. The differential expression between naive and IL-10–stimulated cells was investigated using the interaction term from DESeq2, which enables testing if the expression change (fold change) comparing naive to IL-10 expression levels significantly differed between the WT and IL-10RB^−/−^ Mφs. Similarly, we also tested whether WT and IL-10RB^−/−^ Mφs showed any significant difference in their gene expression changes after LPS or LPS plus *IL10* stimulation. Again, the interaction term from DESeq2 was used to determine any significant differences in fold changes in gene expression. Testing for enrichment of Gene Ontology terms, transcription factor target genes, and metabolic pathways was performed at the InnateDB web server using the default conditions (Breuer et al., 2013). For the network analysis, the interactions for the 21 genes of interest were retrieved from InnateDB, and the network was exported from the network analyst (Xia et al., 2014). The subnetwork was extracted, the expression data (differences of WT vs. mutant in the differential expression between LPS and LPS plus *IL10*) was overlayed, and all visualization was performed in Cytoscape. The prioritized IBD GWAS gene candidates from previously published studies (Jostins et al., 2012; Liu et al., 2015) were used to compare our RNASeq datasets. Clustering based on gene expression profiles was performed using the mfuzz package (Kumar and Futschik, 2007).

### Enrichment of IBD gene loci among IL-10–regulated genes in Mφs

#### IBD loci and genes

The list of IBD genes came from all candidate IBD genes implicated by the prioritization techniques described previously (de Lange et al., 2017; Jostins et al., 2012; Liu et al., 2015). This includes a total of 361 genes across 165 loci. We also used a smaller list of high-certainty IBD genes, consisting of any gene in the previous list that was validated by fine-mapping (de Lange et al., 2017; Huang et al., 2017), plus genes in loci with only one gene implicated by multiple prioritization techniques by Jostins et al. (2012). This list consists of 60 genes across 60 loci.

#### Calculating the number of IBD loci overlapping a gene list

For a given gene list (e.g., the list of differentially expressed genes after LPS stimulation), we calculated the number of IBD loci that contain at least one gene in that gene list. Note that this number is usually smaller than the number of overlapping genes, as often multiple coexpressed genes lie within the same IBD locus.

We compared the number of IBD loci that overlap the gene list with the distribution of overlaps expected under the null, calculated by measuring the number of overlapping loci across 10,000 null gene sets (see below). We calculated the enrichment over null expectation (i.e., the ratio of overlapping IBD loci to the average number of null overlaps in the 10,000 sets), and a one-tailed P value (the proportion of the 10,000 sets that had as many, or more, overlaps with IBD loci than the true value).

#### Null sets

For each gene set, we generated 10,000 null gene sets that were matched for gene expression and gene length. To generate each null set, for each gene in the real gene set, we selected a null gene uniformly from the set of all genes that were within five percentile points of the real gene and added it to the null set. We excluded both the real genes themselves and previously selected null genes in the same null set, to produce a list of unique null genes that did not overlap with the original gene set.

### Data availability

RNASeq data are stored in the European Genome-Phenome Archive under study accession no. EGAS00001001283. Data will be made available to all researchers upon request to the Data Access Committee for the Wellcome Trust Sanger Institute. The restriction on data access is required for human donor protection.

### Online supplemental material

Fig. S1 shows characterization of IL-10RB^−/−^ iPSCs and their ability to differentiate into three germ layers. Fig. S2 compares the cytokine response between IL-10RB^−/−^ and Fpdj_3 Mφs in response to *S.* Typhimurium infection in the presence or absence of IL-10. Fig. S3 compares bacterial killing between IL-10RA^−/−^, IL-10RB^−/−^, and corresponding isogenic controls in the presence or absence of IL-10. Fig. S4 compares the LPS-induced cytokine response between IL-10RA and kolf_2 as well as IL-10RB^−/−^ and IL-10RB^comp^ Mφs in the presence or absence of IL-10. Fig. S5 shows pathway analysis and transcription factor enrichment analysis for LPS- and IL-10–regulated genes. Table S1 shows a list of LPS-inducible genes that are shared between both WT and IL-10RB^−/−^ Mφs. Table S2 shows a list of LPS–down-regulated genes that are shared between both WT and IL-10RB^−/−^ Mφs. Table S3 shows a list of genes up- or down-regulated by IL-10 stimulation alone in WT Mφs but that remain unchanged in IL-10RB^−/−^ Mφs. Table S4 shows a list of LPS-inducible genes that are repressed by IL-10 in WT but not in IL-10RB^−/−^ Mφs. Table S5 shows a list of LPS–down-regulated genes that are rescued by IL-10 in WT but not in IL-10RB^−/−^ Mφs. Table S6 shows a list of all prioritized gene candidates from IBD GWAS. Table S7 lists common genes between IBD GWAS candidates and IL-10–regulated Mφ genes.

## Supplementary Material

Table S1
